# Improved pyrrolysine biosynthesis through phage assisted non-continuous directed evolution of the complete pathway

**DOI:** 10.1038/s41467-021-24183-9

**Published:** 2021-06-24

**Authors:** Joanne M. L. Ho, Corwin A. Miller, Kathryn A. Smith, Jacob R. Mattia, Matthew R. Bennett

**Affiliations:** 1 Department of Biosciences, Rice University, Houston, TX USA; 2 Department of Bioengineering, Rice University, Houston, TX USA

**Keywords:** Synthetic biology, Metabolic engineering, Molecular evolution, Applied microbiology

## Abstract

Pyrrolysine (Pyl, O) exists in nature as the 22^nd^ proteinogenic amino acid. Despite being a fundamental building block of proteins, studies of Pyl have been hindered by the difficulty and inefficiency of both its chemical and biological syntheses. Here, we improve Pyl biosynthesis via rational engineering and directed evolution of the entire biosynthetic pathway. To accommodate toxicity of Pyl biosynthetic genes in *Escherichia coli*, we also develop Alternating Phage Assisted Non-Continuous Evolution (Alt-PANCE) that alternates mutagenic and selective phage growths. The evolved pathway provides 32-fold improved yield of Pyl-containing reporter protein compared to the rationally engineered ancestor. Evolved PylB mutants are present at up to 4.5-fold elevated levels inside cells, and show up to 2.2-fold increased protease resistance. This study demonstrates that Alt-PANCE provides a general approach for evolving proteins exhibiting toxic side effects, and further provides an improved pathway capable of producing substantially greater quantities of Pyl-proteins in *E. coli*.

## Abstract (web summary)

Pyrrolysine (Pyl) exists in nature as the 22^nd^ proteinogenic amino acid, but studies of Pyl have been hindered by the difficulty and inefficiency of both its chemical and biological syntheses. Here, the authors developed an improved PANCE approach to evolve the pylBCD pathway for increased production of Pyl proteins in *E. coli*.

## Introduction

Pyrrolysine (Pyl, O) exists in nature as the 22nd proteinogenic amino acid^1^. Pyl represents an ancient addition to the genetic code, believed to have been present in the last universal common ancestor^2^. Today, Pyl is found in numerous bacterial and archaeal species but not in eukaryotes. Although Pyl has been found in several classes of proteins^3^, it is best known for its essential role in a unique class of methanogenic enzymes^1,4^. Pyl has a remarkably distinct structure compared to other proteinogenic amino acids and is noteworthy for its reactive electrophilic moiety^1^—a feature absent in all other proteinogenic amino acids.

**Fig. 1:**
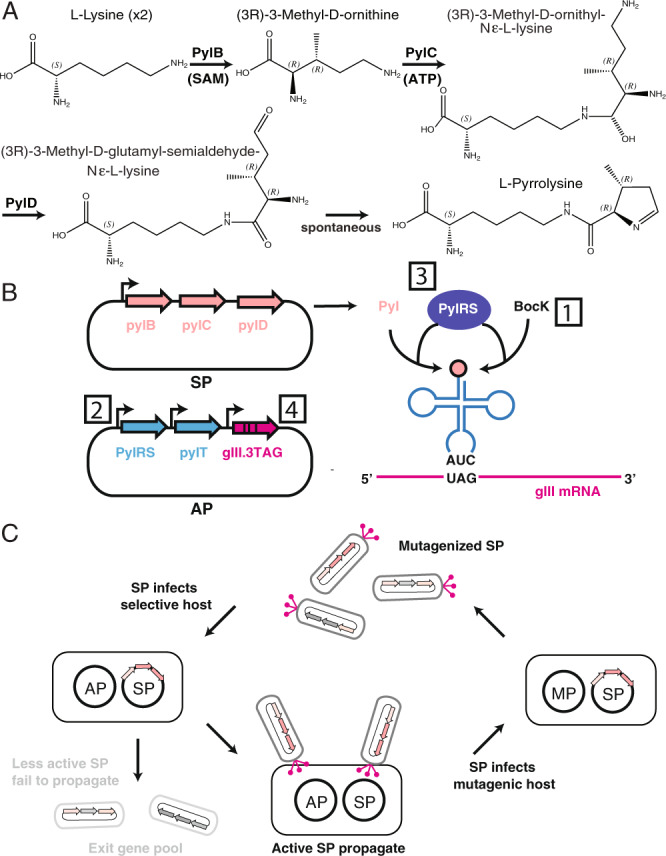
Pyl biosynthetic pathway evolution via Alt-PANCE. **A** Pyl biosynthesis involves condensation of two lysine molecules by three enzymes—the radical SAM enzyme PylB, ATP-dependent PylC, and PylD. **B** Biosynthetic *pylBCD* operon was cloned into a selection phage (SP), while the constitutively expressed *pylST* and phage shock promoter-controlled *gIII* were cloned into accessory plasmid (AP) vectors. Numbered portions indicate selection circuit elements that were altered throughout evolution to control selection stringency, including (1) supplementation with BocK, (2) expression level of PylRS, (3) mutant variants of PylRS used, and (4) the number of amber codons present in *gIII*. **C** Each Alt-PANCE round entailed two phage passages: selective passage (left) entails operon activity-dependent expression of PIII (pink rods with circular tips); mutagenic passage (right) entails mutagenesis plasmid (MP)-dependent mutagenesis of the SP.

The genetic components required for Pyl incorporation are encoded in a single operon, *pylSTBCD*^4^, which mediates Pyl biosynthesis and protein incorporation through nonsense suppression of amber (UAG) codons^5^. Within the operon, *pylS* encodes pyrrolysyl-tRNA synthetase (PylRS), which catalyzes the ligation of Pyl to tRNA, while *pylT* encodes the corresponding transfer RNA (tRNA^Pyl^)^4^. Genes *pylB*, *pylC*, and *pylD* encode enzymes that biosynthesize pyrrolysine from lysine (Fig. 1A)^4^. To date, numerous genetic code expansion studies have utilized PylRS and tRNA^Pyl^ to incorporate synthetic amino acids into proteins, as these genes provide an aminoacyl-tRNA synthetase (aaRS)-tRNA pair that does not exhibit cross-reactivity with the existing *Escherichia coli* translation system^6–10^.

In contrast to most synthetic amino acids, Pyl is naturally recognized by PylRS and is ligated to tRNA^Pyl^ with high efficiency. However, studies of Pyl proteins have been hindered by the poor supply of the amino acid despite being a fundamental building block of proteins in nature. An increased level of Pyl are thus needed to further our understanding of these proteins. Improving production of Pyl proteins provides an unusual challenge, as genetic code expansion studies have typically focused on improving aaRS-tRNA pairs for better recognition of synthetic amino acids. To date, Pyl protein production has been severely limited by the poor activity of the archaeal biosynthetic *pylBCD* pathway. When this pathway is expressed heterologously in laboratory strains (such as *E. coli*), Pyl proteins are produced at a very low yield^4,11^. An alternative to improving biosynthesis is to supply cells with an exogenous source of Pyl^12^. However, organic synthesis of Pyl is known for its difficulty^12–14^ and it remains commercially unavailable.

We anticipated that Phage-Assisted Non-Continuous Evolution (PANCE)^15^ would provide an effective method for improving production of Pyl proteins in *E. coli*, as Pyl biosynthesis is conducted by three genes across which problematic regions are difficult to identify. In phage-assisted evolution, the activity of an evolving gene of interest is linked to the life cycle of M13 bacteriophage, allowing each generation of phage growth to effectively serve as a cycle of directed evolution^16^. PANCE, which relies on serial flask transfers, and its chemostat-based counterpart PACE have previously been used to rapidly evolve increased activity in a wide variety of individual enzymes, including RNA polymerases^16^, proteases^17^, and aminoacyl-tRNA synthetases^18^. Of note, PANCE is categorized as an in vivo continuous-directed evolution method^19^; “non-continuous” within its name denotes its use of serial culture transfers, in contrast to the continuous flow machinery used for PACE^16^. Contrary to more targeted approaches (such as saturation mutagenesis), PANCE allows an entire genetic region to be quickly evolved without focusing on specific regions of interest^20^.

Here, we detail the improvement of the *pylBCD* pathway for increased production of Pyl proteins in *E. coli*, performed using a two-step process. Our first step entails the rational addition of a solubility tag to *pylB*, resulting in reduced toxic protein aggregation within the cell and also facilitating detectable levels of Pyl-containing sfGFP production. We next devise a version of PANCE that we term Alternating PANCE (Alt-PANCE), designed to accommodate mild to moderate cellular toxicity during evolution. We use this method to evolve *pylBCD* for increased activity across numerous selection conditions. This process result in an additional 32-fold increase in Pyl-sfGFP production mediated by our most active mutant. Our evolutionary characterization find that the majority of mutations occurred within *pylB*, and serve to increase cellular accumulation of this protein by ~4–5-fold and increase its in vitro protease resistance by ~2-fold. This work provides both a procedure to enable continuous directed evolution of proteins exhibiting toxic side effects, and further provides a substantially improved biosynthetic pathway for bacterial production of Pyl proteins.

## Results

### Devising Alt-PANCE and improving PylB solubility

We initially attempted to use PANCE to evolve a codon-optimized variant of the *M. acetivorans pylBCD* pathway, and the poor initial activity of these genes led us to perform additional optimization before beginning evolution. Following overexpression of the *pylBCD* pathway, we observed formation of inclusion bodies within each cell (Supplementary Fig. 1). After noting that cells expressing only *pylCD* did not form inclusion bodies, we rationally fused a SUMO tag to the N terminus of PylB to improve its solubility^21^. The addition of a SUMO tag has previously been shown to improve PylB solubility, enabling purification and crystallization of this protein^22^. Following the addition of a SUMO tag to *pylB*, we observed that expression of SUMO-*pylBCD* resulted in healthy cells without inclusion bodies, indicating improved PylB solubility in vivo and reduced toxic side effects (Supplementary Fig. 1). We next cloned SUMO-*pylBCD* into an M13 selection phage (SP) vector, termed SP.BCD (see “Methods”).

As expression of SUMO-*pylBCD* still exhibited a moderate toxic effect on *E. coli* cells, we next developed an alternating version of PANCE^15^ that we termed Alt-PANCE (Fig. 1C) to enable evolution of this pathway for improved activity. Typically, PANCE exposes evolving phage to simultaneous selection and mutagenesis, both of which lead to a high fitness cost and reduced phage titers. We further observed that expression of *pylBCD* in *E. coli* results in an additional fitness cost, the cumulative effect of which precludes simultaneous selection and mutagenesis (Supplementary Fig. 2A). In Alt-PANCE, alternating passages are performed in which evolved phage are grown while subjected to mutagenesis only (without activity selection), followed by selection only (without mutagenesis). The Alt-PANCE procedure was thus developed to reduce the fitness cost associated with continuous evolution of genes exhibiting mild to moderate toxicity. Here we use Alt-PANCE to evolve the entire Pyl biosynthetic pathway, demonstrating the second instance of simultaneous PACE co-evolution of multiple genes, following the recent report on evolution of bicyclomycin biosynthetic genes^23^.

To mediate genetic selection, we expressed *pylS* and *pylT* within cells to link Pyl production to translation of an amber mutant of the essential phage gene, *gIII*^16^. Following Pyl biosynthesis, PylRS ligates Pyl to tRNA^Pyl^, which leads to Pyl incorporation at 1–3 amber codons within *gIII* and expression of functional PIII (Fig. 1B). Besides mediating selection, covalent ligation of Pyl to tRNA^Pyl^ also limits cell-to-cell diffusion of Pyl, which facilitates evolution by reducing evolutionary “cheating”. A similar selection system was used in previous work to evolve *pylS* for improved incorporation of a Pyl analog—*N*ɛ-Boc-l-lysine (BocK)^15,18^. Like Pyl, this synthetic amino acid mediates amber suppression^15^.

### Continuous directed evolution of Pyl biosynthesis pathway

Following our engineered improvement of PylB solubility, the *pylBCD* pathway exhibited sufficient activity to initiate Alt-PANCE. We began evolution at low selection stringency by supplementing the media with a starting concentration of BocK (200 µM) high enough to ease phage propagation yet low enough that phage variants producing more Pyl had a selective advantage (Supplementary Fig. 2B). During selection growths, we began by using accessory plasmid (AP) JH61, which encodes *gIII* containing one amber codon and a highly expressed PylRS (Supplementary Table 1). During mutagenic growths, phage were propagated using strain S1059 (ref. ^24^), a permissive strain that expresses *gIII* following phage infection without imposing a selection. Mutagenic growths also included the presence of mutagenesis plasmid MP6, previously shown to increase the mutation rate by five orders of magnitude following arabinose induction^25^ (Supplementary Fig. 2A). SP.BCD was capable of separately propagating across both the selective and mutagenic conditions described above, thereby enabling Alt-PANCE initiation.

We performed Alt-PANCE of SP.BCD across three independent lineages, termed lineages A, B, and C. As phage continued to evolve, they were passaged across cells exhibiting a total of 11 different stringency conditions, with each successive change predicted to increase selection strength through variation of four separate parameters (Fig. 1B and Supplementary Table 1). After each round of Alt-PANCE, we tested phage growth under stronger selection conditions; we then either maintained selection strength at the prior level in the subsequent round, or increased it if growth was possible. Initially, we increased selection strength by decreasing BocK supplementation, thereby forcing evolving phage to produce additional Pyl to fill the gap. Once the evolving phage could propagate in the absence of BocK, we increased selection strength by altering the AP vector via three approaches: (1) we reduced expression of *pylS* by mutating its promoter and ribosome-binding site (RBS), (2) we increased the number of amber codons in *gIII*, and (3) we substituted mutant *pylS* variants with reduced Pyl affinity^15^. Each of these approaches required evolving phage to produce greater amounts of Pyl in order to maintain comparable levels of *gIII* translation. We halted evolution upon reaching the final stringency condition after 34–40 rounds of Alt-PANCE (Supplementary Figs. 2C and 3).

### Analysis of evolved *pylBCD* mutations

**Fig. 2:**
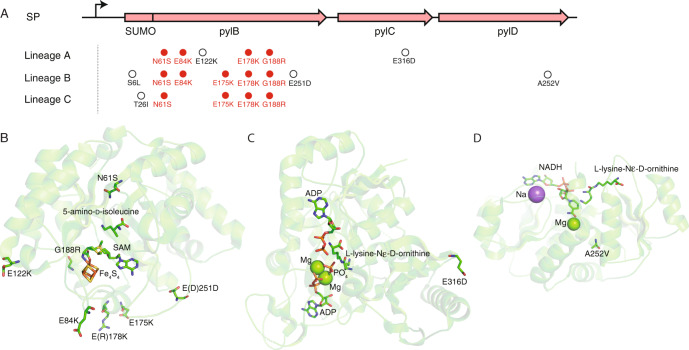
Convergent mutations in the co-evolved *pylBCD* genes. **A** Locations of critical mutations identified in three subpopulations shown within the previously solved structures of *M. barkeri* **B** PylB (PDB: 3T7V), **C** PylC (PDB: 4FFP), and **D** PylD (PDB: 4J4B); amino acid substitutions were all found outside the enzyme active sites, with many PylB mutations resulting in increased cationic surface charge. Numbering scheme is based on the wild-type protein sequences of PylB, PylC, and PylD in *Methanosarcina acetivorans*. Specifically, numerical labels for PylB refer to the WT PylB protein sequence and do not include the 104 amino acids added by the SUMO tag. Brackets indicate residues naturally occurring in *M. barkeri* proteins that have diverged from the *M. acetivorans* sequence. Mutations highlighted in red were observed to be convergent across multiple subpopulations (for further mutation analysis of subpopulations, see Supplementary Tables 2 and 3).

For each lineage, we isolated 10–15 phage plaques; for each isolate, we sequenced the *pylBCD* insert, its upstream promoter, and ribosome-binding sites. We identified 5–8 mutations in each lineage (Fig. 2A and Supplementary Table 2), 11 convergent high-frequency mutations (Supplementary Fig. 3 and Supplementary Table 2), and a total of 16 unique mutations (Supplementary Table 2). All mutations were found within protein coding sequences and the majority were distal to the active site of each enzyme (Fig. 2B). We identified six distinct subpopulations—36A_sub-pop1, 36A_sub-pop2, 34B_sub-pop3, 34B_sub-pop4, 34B_sub-pop5, and 40C_sub-pop6—that contained representative combinations of convergent mutations (Supplementary Table 3). The *pylBCD* cassettes from these subpopulations were cloned into expression vectors to measure the activity of each variant via a coupled super-folder green fluorescent protein (sfGFP) reporter assay, wherein biosynthesized Pyl is incorporated into sfGFP though amber suppression (see “Methods”).

**Fig. 3:**
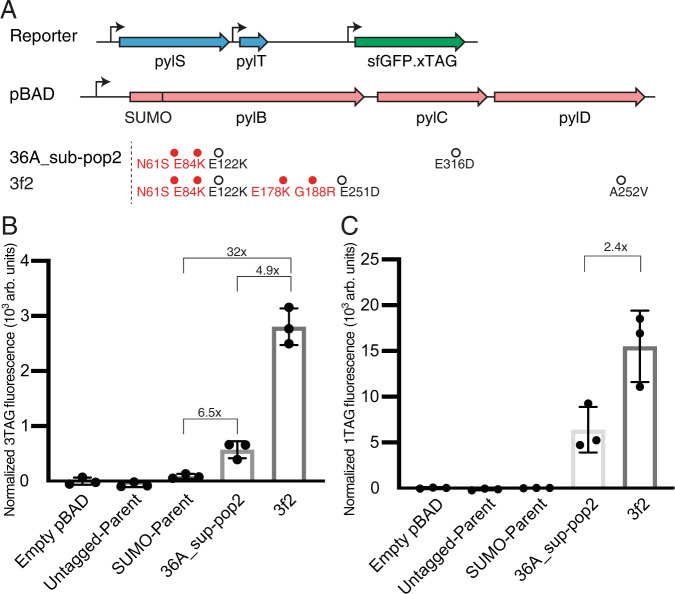
Evolved biosynthetic pathway mediates improved Pyl protein yield. **A** The best evolved variant (36A_sub-pop2) and best combinatorial variant (3f2) contained a total of four and seven mutations, of which two and four were convergent mutations (red), respectively (for further mutation analysis of subpopulations, see Supplementary Tables 2 and 3). **B** When tested in *E. coli* strain C321.∆A.exp, read-through of three amber codons in sfGFP is improved 32-fold in variant 3f2 combinatorial variant and 6.5-fold in variant 36A_sub-pop2, relative to the parental variant SUMO-*pylBCD*. The WT *pylBCD* pathway (termed “Untagged Parent”) did not have detectable activity. **C** Read-through of one amber codon in sfGFP is improved 2.4-fold in variant 3f2, relative to variant 36A_sub-pop2. The WT *pylBCD* variant and the SUMO-*pylBCD* did not have detectable activity in this assay. In panels **B** and **C**, fluorescence intensity shown (excitation 488 nm, emission 509 nm) was normalized by optical density (*A*_600_) (see “Methods”). Samples were tested in biological triplicate; data shown represents mean values ± s.d.

While most evolved mutants appeared to exhibit higher activity than the ancestral variant SUMO-*pylBCD*, the highest activity levels were observed in 36A_sub-pop1, 36A_sub-pop2, and 34B_sub-pop3 (Supplementary Fig. 4). Next, we rationally combined mutations originating from separate lineages to produce five combinatorial variants (Supplementary Table 3) and observed that 3f2 and JM10.1 exhibited the highest activity (Supplementary Fig. 5). Although ancestral variant SUMO-*pylBCD* did not produce detectable sfGFP signal under other assay conditions, activity was observed when mediating suppression of three amber codons in *E. coli* strain C321.∆A.exp^26^ (see “Methods”). Strain C321.∆A.exp was chosen for these experiments as it has previously been engineered for improved ncAA incorporation at amber codons, as RF1 has been deleted in this strain and all genomic instances of amber codons have been recoded. Under these conditions, variant 3f2 exhibited 4.9-fold greater activity compared to variant 36A_sub-pop2, and 32-fold greater activity compared to ancestral variant SUMO-*pylBCD* (Fig. 3). The WT *pylBCD* variant did not produce detectable sfGFP activity under any condition tested. However, luminescent signal was observed following expression of this variant in a separate coupled luciferase activity assay (Supplementary Fig. 6, see “Methods”). The luciferase assay found that SUMO-BCD had 7.8-fold greater luminescent activity compared to the untagged WT parent, evolved variant 36A_sub-pop2 exhibited 1.6-fold greater activity compared to SUMO-BCD, engineered variant 3f2 exhibited 1.7-fold greater activity compared to 36A_sub-pop2, and engineered variant 3f2 exhibited 21-fold greater activity compared to the untagged WT parent. His-tagged sfGFP containing two amber codons was produced using variant 3f2, purified, and analyzed by liquid chromatography tandem mass spectrometry (LC-MS/MS) to confirm the identity of Pyl at positions 39 (Supplementary Fig. 7 and Supplementary Table 4) and 151 (Supplementary Fig. 8 and Supplementary Table 5, see “Methods”).

Assessing the 16 unique mutations identified within the *pylBCD* operon at the endpoint, the majority (9) were found within the *pylB* coding sequence, and two of those were within the SUMO tag (Supplementary Table 2). Each isolated subpopulation contained significantly more mutations in *pylB* than in *pylC* or *pylD*, with two subpopulations (36A_sub-pop1 and 40C_sub-pop6) containing no mutations in *pylC* or *pylD*. This disparity is also observed in the highly active combinatorial variants 3f2 and JM10.1. Those two variants each contained only a single mutation in *pylD*, no mutations in *pylC*, and either six or eight mutations in *pylB* (Supplementary Table 3). These observations suggest that improved Pyl production primarily stemmed from mutations in *pylB*, consistent with prior biochemical evidence and quantum mechanical simulations indicating this enzyme catalyzes the rate-limiting step of Pyl synthesis^11,27^.

### Characterization of PylB mutants

Notably, *pylB* mutations were remarkably convergent in character, collectively increasing the cationic charge of the protein surface. Each Alt-PANCE evolved subpopulation contained mutations that increased the charge of SUMO-PylB by +2 to +5. Combinatorial variants 3f2 and JM10.1 exhibited an even greater change, with cationic charge increasing by +7 and +9, respectively. Mutations changing anionic glutamic acid to cationic lysine residues were prevalent, with four separate instances observed (E84K, E122K, E175K, and E178K). Analysis of the crystal structure of PylB from a closely related organism *Methanosarcina barkeri*^22^ revealed that all four E-to-K mutations occurred at solvent-exposed regions of the protein surface (Fig. 2B). This pattern suggests that, instead of or in addition to directly improving catalytic properties of PylB, these mutations may confer a benefit towards the biophysical properties of the protein, thereby improving PylB stability within the cell.

**Fig. 4:**
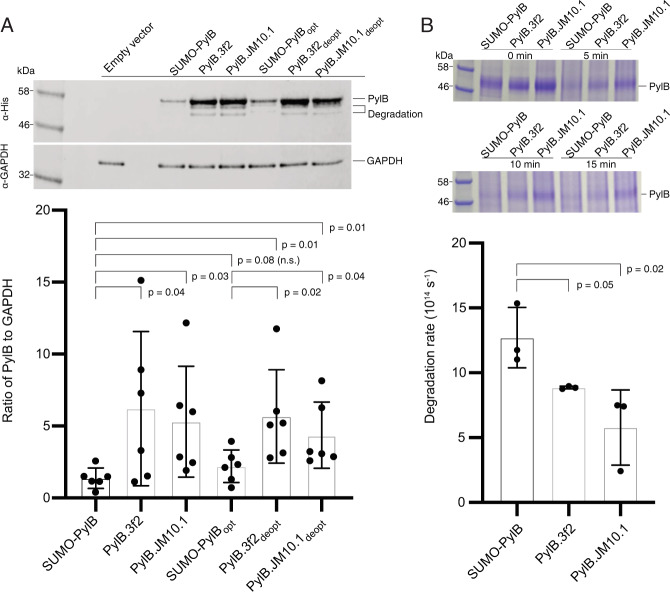
Evolved PylB mutants are present at elevated levels inside cells, and show increased protease resistance. **A** Quantitative western blot assays were performed on clarified lysates of cells expressing different His-tagged PylB variants. Prior to cell lysis, cultures all found to exhibit similar OD_600_ values (3.1–3.3), colony-forming units (1–3 × 10^9^ CFUs/mL), and cell pellet wet weights (1.2–1.4 g). For each sample, His-tag antibodies were used to quantify PylB, and samples were normalized using the housekeeping gene GAPDH (see “Methods”). These experiments show two evolved mutants, PylB.3f2 and PylB.JM10.1, are present at a higher concentration compared to the ancestral variant SUMO-PylB. These results further show that two codon-deoptimized evolved mutants, PylB.3f2_deopt_ and PylB.JM10.1_deopt_, are also present at higher concentrations compared to the codon-optimized ancestral variant SUMO-PylB, suggesting that the elevated levels of evolved mutant proteins likely do not results from improved codon usage. **B** Proteolytic degradation assays were performed using purified PylB variants. Protein samples were incubated with the protease enzyme chymotrypsin, and the concentration of PylB was evaluated over time (see “Methods”). Evolved mutants PylB.3f2 and PylB.JM10.1 were found to degrade at a slower rate compared to the ancestral variant SUMO-PylB, indicating increased protease resistance in the evolved mutants. Samples were tested in biological sextuplicate; data shown represent mean values ± s.d. For both figures **A** and **B**, *p* values were calculated using a one-tailed Student’s *t*-test, without adjustments for multiple comparisons. Source data are provided as a Source Data file.

To better characterize evolved mutations in PylB, we next overexpressed and purified two evolved SUMO-PylB variants (from 3f2 and JM10.1 cassettes) as well as the ancestral SUMO-PylB for further analysis (see “Methods”). Consistent with prior work, we were unable to measure activity from any purified PylB samples, owing to the extreme lability of its SAM cofactor^22^. However, during purification we noted that protein yields of both evolved PylB variants were substantially greater than that of the ancestral SUMO-PylB (Supplementary Fig. 9). After repeating each purification in triplicate, we found that variants PylB.3f2 and PylB.JM10.1 producing 6.0- and 5.6-fold greater yields compared to the ancestral variant, respectively (Supplementary Fig. 10). To confirm these findings, we next performed quantitative western blot assays on clarified cell lysates, using α-His antibodies to detect His-tagged PylB and α-GAPDH antibodies to detect the housekeeping gene GAPDH (see “Methods”). After using GAPDH concentrations to normalize PylB levels in each sample, we found that evolved variants PylB.3f2 and PylB.JM10.1 accumulated at 4.5- and 3.8-fold higher concentration (respectively) compared to the ancestral variant SUMO-PylB (Fig. 4A).

In light of these results, we next sought to investigate the mechanisms underlying the increased protein levels observed in our evolved mutants. As our PylB variants were each cloned into identical expression constructs, most factors dictating protein expression levels (such as promoter and ribosome-binding site strength) will be the same for each mutant. We thus considered whether more efficient codon usage could explain the increased yields, and began by comparing the *E. coli* codon usage frequency^28^ of each evolved and ancestral codon at mutated PylB positions (Supplementary Table 6). In this analysis, we found that the vast majority of PylB mutations resulted in less frequently used codons, hence our evolved PylB variants would be predicted to be translated less efficiently than the ancestral variant SUMO-PylB. To further explore the effects of codon usage, we next prepared a construct expressing an ancestral PylB variant containing numerous silent changes at evolved mutation sites designed to further optimize codon usage (termed SUMO-PylB_opt_). We also prepared two constructs expressing evolved PylB variants designed to deoptimize codon usage at evolved mutations sites, termed PylB.3f2_deopt_ and PylB.JM10.1_deopt_ (Supplementary Table 6). We included these three mutants in our quantitative western blot experiments, and found that codon optimization or deoptimization had a relatively small effect on protein levels, with PylB.3f2_deopt_ and PylB.JM10.1_deopt_ found to be expressed at 2.6- and 2.0-fold higher levels (respectively) compared to SUMO-PylB_opt_ (Fig. 4A). Taken together, these results suggest that elevated levels of evolved PylB mutants largely cannot be attributed to more efficient codon usage, and are instead more likely explained by changes in protein properties.

We next considered protein properties that might improve the lifespan of evolved PylB mutants within the cell. As altered protein surface charge is associated with changes in solubility^29^, and given that activity improvements were previously achieved through addition of a SUMO solubility tag, we performed precipitation experiments to evaluate the solubility of our purified PylB samples. While no precipitation was observed following exposure to the precipitant ammonium sulfate, exposure to PEG-8,000 induced aggregation in each PylB sample (see “Methods”). Noting that protein surface charge has also been associated with adaptation to different salt conditions^29^, we performed separate precipitation experiments under both high-salt (250 mM NaCl) and low-salt (10 mM NaCl) conditions. Following exposure to varying amounts of PEG-8,000, both evolved mutants exhibited a greater propensity to aggregate compared to the SUMO-PylB ancestor, indicative of reduced solubility under both high- and low-salt conditions (Supplementary Fig. 11). We further confirmed that solubility is not improved within evolved mutants 3f2 and JM10.1 by cloning SUMO tag deletion variants, which produced inclusion bodies following pathway induction (Supplementary Fig. 12).

We next examined the effects of evolved PylB mutations on protein thermostability, as protein stability is a key determinant of steady-state protein concentration^30^. For these experiments we used differential scanning fluorimetry (DSF, also known as the Themofluor assay)^31^, a method in which a fluorescent probe is used to monitor protein unfolding as samples are slowly heated (see “Methods”). This assay was performed using each of the aforementioned purified PylB samples, first testing low- and high-salt conditions (Supplementary Fig. 13 and Supplementary Table 7) and subsequently testing medium-salt conditions with the addition of PylB cofactor SAM, the addition of PylB substrate l-lysine, the addition of both additives, and the addition of neither (Supplementary Fig. 14 and Supplementary Table 8). While each of these conditions did lead to marginally altered Tm values, in each case the mutant proteins did not show significantly greater thermostability compared to the ancestral variant.

We lastly tested the susceptibility of our PylB variants to proteolysis, as improved protease resistance can reduce the rate of protein degradation inside cells^32^. Protease resistance has long been used as a metric for protein stability, as less stable proteins tend to more readily unfold and become more accessible to proteolytic cleavage^33,34^. For our experiments, we used the protease chymotrypsin. Chymotrypsin is a relatively nonspecific protease, cleaving protein substrates after Trp, Tyr, Phe, or Leu residues. As our evolved mutations in PylB do not affect chymotrypsin cut sites, using this protease avoids biasing our assays by affecting the number of cleavage sites between mutants. In these assays, we incubated our purified PylB variants in vitro with chymotrypsin and measured the amount of PylB remaining over time (see “Methods”). We observed that the rate of degradation for the ancestral variant SUMO-PylB was 1.4-fold greater compared to PylB.3f2, and 2.2-fold greater compared to PylB.JM10.1 (Fig. 4B). This finding provides a potential mechanistic explanation for the increase concentrations of our evolved PylB mutants observed inside cells, suggesting they have reduced rates of protein degradation.

## Discussion

In light of our above results, we thus conclude that *pylBCD* mutations evolved during Alt-PANCE mediate improved Pyl protein production by increasing steady-state concentrations of PylB. While altered catalytic activity cannot be ruled out, the ~5-fold increase in PylB concentration within evolved mutants may largely account for the ~32-fold increase in Pyl-sfGFP production (Fig. 3), as an increase in enzyme concentration will produce an even larger fold increase in product formation. Regarding the mechanism underlying these increased PylB yields, our results indicate that factors dictating protein production rate (such as optimal codon usage) likely do not play a significant role. Our experiments instead suggest reduced rates of PylB degradation as a potential mechanism for the elevated protein levels of our PylB mutants, as our evolved mutants were observed to be more protease resistant. As susceptibility to proteolytic degradation is associated with sampling of unfolded states, our results may indicate that the evolved PylB mutants more readily adopt a correctly-folded conformation.

We note here that we also attempted multiple strategies to extract and quantify free Pyl following its biosynthesis (see “Methods”). Although our approach was guided by prior work^35^, we were unable to detect Pyl in our numerous extracts. While the reasons underpinning our inability to reproduce this protocol are unclear, we postulate that our efforts may have been complicated by chemical modification or degradation of the free amino acid within the complex cellular mileu. Indeed, similar instability is observed with the twenty-first amino acid selenocysteine, as this residue and its biosynthetic intermediates are highly labile; in fact, selenocysteine becomes stabilized only upon incorporation into proteins^36,37^.

The Alt-PANCE procedure developed here establishes a general methodology for rapidly evolving proteins for improved function while acommodating toxic side effects. Our results indicate this method can improve the weakest link within an evolving pathway by altering biophysical parameters that are difficult to address via rational or targeted methods^38^. While prior PACE and PANCE experiments have primarily evolved single proteins^19,39^, here we apply these techniques to a complete multi-gene biosynthetic pathway. A persistent challenge lay in diffusion of biosynthesized products between competing cells^39^, which enables evolutionary escape. Our results demonstrate that ligation of the biosynthetic product to tRNA sufficiently reduces diffusion to enable successful continuous directed evolution. As numerous naturally occurring ncAAs cannot be biosynthesized in *E. coli* at high yield^40^, our selection system could be applied to evolve other valuable amino acid biosynthetic pathways.

Our improved *pylBCD* pathway enables production of useful Pyl proteins at substantially greater yields. As the 22nd amino acid, Pyl is fundamental to the origins of life, yet has long been difficult to study. In nature, Pyl is a critical residue in methanogenic enzymes—*mttB*, *mtbB, mtmB*—that produce methane from methylamines^1^. Methane is increasingly viewed as a substitute for fossil fuels, but microbial methanogenesis is only known to occur in archaea^41^, which is recalcitrant to genome engineering. Heterologous expression of these Pyl-containing methanogenic enzymes in *E. coli*, a chassis much easier to engineer than archaea, is now feasible and may help towards the production of industrially relevant quantities of methane, though significant additional effort would still be required.

The unusual chemical properties of Pyl further enable other exciting bioengineering applications. While electrophilic moieties are notably absent in other proteinogenic amino acids^4^, the imine group found within the Pyl pyrrole ring is both electrophilic and highly reactive. This moiety has been previously shown to react with 2-amino-benzaldehyde (2-ABA) and 2-amino-acetophenone (2-AAP) groups to form a bio-orthogonal crosslink^11^, which provides a bioconjugation method with unique chemistry distinct from other approaches. The positive charge carried by Pyl also enables its use in antimicrobial peptides whose mechanisms of action require a cationic charge^42^. Additionally, considering the atypical structure of Pyl, peptides containing this residue are likely to resist proteolytic digestion^43^. Pyl is thus unusually well suited for use in antimicrobial peptides, as degradation remains the primary hurdle for their therapeutic application. This work achieves the facile overproduction of Pyl-containing proteins, enables use of this remarkable chemical in future studies, and provides insight on the biophysical properties of the biosynthetic enzymes that produce this understudied naturally occurring non-canonical amino acid.

## Methods

### Alt-PANCE of *pylBCD* pathway

One round of Alt-PANCE was performed each day; each round consisted of two passages—one mutagenic (16 h) and one selective (8 h). For mutagenic passages, *E. coli* strain S1059 cells carrying plasmid MP6 were used as the host; 10 mM l-arabinose was added to the culture to induce MP6 immediately following the addition of phage. Activity of MP6 was tested by separate serial spotting of mutagenic host cells onto glucose and arabinose containing agar (see Supplementary Fig. 2A). For selective passages, *E. coli* strain S1030 cells carrying an AP were used as the host (for specific APs used at each transfer, see Supplementary Table 1). Test outgrowths were performed at higher stringency conditions after each selective passage. Nε-Boc-l-lysine (BocK) (Chem-Impex, cat. # 00363) was added during initial selective passages to tune selection strength (see Supplementary Table 1). Ramps in stringency condition of S1030/AP are as shown in Supplementary Fig. 2C. Three lineages (Lineages A–C; Fig. 2 and Supplementary Fig. 3) were independently evolved. *E. coli* host cell was grown in 2xYT media supplemented with 20 mM glucose and 5 mM magnesium chloride in a 1 L baffled flask, at 37 °C with shaking at 225 r.p.m. until reaching *A*_600_ = 0.3−0.5, whereupon the culture was stored at 4 °C for up to a week, for bacteriophage transfection experiments; bacteriophage transfection was performed by transferral of at least 10^6^ phage-forming units from a previous growth culture into a fresh 50 mL host cell culture^15^. Transfected cells were grown at 37 °C with shaking at 225 r.p.m. for 12 h. Phage produced following each passage were quantified by phage titer and stored at 4 °C.

### Molecular cloning

The *M. acetivorans* Pyl biosynthetic pathway (NCBI locus tags MA_RS00810, MA_RS00815, and MA_RS00820) was codon-optimized for expression in *E. coli*, and a ribosome-binding site (SD4) was added upstream of *pylC* as the natural ribosome-binding site lies within the coding region of *pylB* (see Supplementary Data 1 for DNA sequences). This sequence was synthesized as an IDT gBlock, and the synthetic DNA was subsequently cloned into pCDF. All other DNA fragments were prepared by PCR. Selection phage (SP) vectors were cloned by inserting DNA into M13 bacteriophage, replacing its essential gene *gIII* to enable survival-mediated selection^16^. Combinatorial mutants were prepared via Gibson assembly and QuikChange mutagenesis; all other vectors were cloned using Gibson assembly. We deposited a set of plasmids, along with maps and sequences, in Addgene (Supplementary Table 6). S1059, S1030, selection phage scaffold, and mutagenesis plasmids were generous gifts from the David Liu lab at Harvard University. The aaRS variants were generous gifts from the Dieter Söll lab at Yale University.

### Fluorescence-based *pylBCD* activity assay

We performed a fluorescence-based readthrough assay that relies on Pyl-tRNA^Pyl^-mediated suppression of amber codons encoded in sfGFP mRNA, producing fluorescence signal measured in a 96-well U-bottom microplate (Falcon) format using a Spark multimode microplate reader (Tecan)^44^. We prepared BL21 (DE3) and C321.∆A.exp strains containing a reporter vector encoding constitutively expressed PylRS, tRNA^Pyl^, and AraC, as well as sfGFP.3TAG (N39O, N135O, and Y151O) under the control of the Para promoter. This strain was transformed with a producer vector encoding either the wild-type *pylBCD* variant or an engineered variant under the control of the PT7/Lac promoter. Cells were grown to *A*_600_ 0.2–0.5, expression of the biosynthetic cassette was induced with 10 µM IPTG for 2 h, and expression of the reporter sfGFP.3TAG protein was induced with 1.3 mM l-arabinose for 3–18 h. Fluorescence intensity (excitation 488 nm, emission 509 nm) was normalized by optical density (*A*_600_) and the best producer was identified. Experiments were performed on biological triplicates and background correction was performed by subtracting the Fl/OD value of the strain transformed with an empty producer vector. Concentrations of IPTG and l-arabinose used in the above protocol were found to result in the highest amount of fluorescent signal after empirically testing additional concentrations.

### Luminescence-based *pylBCD* activity assay

We performed a luminescence-based readthrough assay that relies on Pyl-tRNA^Pyl^-mediated suppression of an amber codon encoded in luxB mRNA, producing luciferase activity measured in a 96-well U-bottom microplate (Falcon) format using a Spark multimode microplate reader (Tecan)^18^. We prepared strain C321.∆A.exp cells containing a reporter vector encoding constitutively expressed *chPylS* and pylT, as well as *luxCDABE* wherein *luxB* contained an amber stop codon after the initiator methionine. This strain was transformed with a producer vector encoding a pylBCD natural or combinatorial variant under the control of the Para promoter. Cells were grown to *A*_600_ 0.2–0.5, and expression of the biosynthetic cassette was induced with 1.3 mM l-arabinose for 3–18 h. Luminescence intensity was normalized by *A*_600_. Experiments were performed on biological triplicates and background correction was performed by subtracting the LUMI/OD value of the strain transformed with an empty producer vector.

### Purification and LC-MS/MS of the Pyl-containing sfGFP

Purification of the Pyl-containing sfGFP variants was enabled via a C-terminal His-tag. Reporter protein was expressed for 24 h at 25 °C in BL21 (DE3) encoding the reporter vector and best producer vector. Protein was purified using a Ni-NTA column, run on a sodium dodecyl sulfate polyacrylamide gel electrophoresis (SDS-PAGE) gel, and the excised 28 kDa band was in-gel digested with chymotrypsin or Asp-N (Roche Diagnostics). LC-MS/MS analysis of digested peptides was performed on an LTQ Orbitrap XL (Thermo Scientific) equipped with a nanoACQUITY UPLC system (Waters), at the Yale Proteomics Center^45^. Trapping was performed for 1 min at 15 µL/min in Buffer A (0.1% formic acid in water). Peptide separation was performed at 300 nL/min using Buffers A and B (0.1% formic acid in CH_3_CN). The linear gradient (51 min) was from 5 to 50% B at 50 min, to 85% B at 51 min. MS data were acquired in the Orbitrap with one microscan, and a maximum inject time of 900 ms followed by data-dependent MS/MS acquisitions in the ion trap (through collision-induced dissociation). The Mascot search algorithm (Matrix Science) was used to search for all amino acid substitutions.

### Light microscopy of *E. coli* cells

*E. coli* containing pCDF plasmids encoding the SUMO-tagged or untagged *pylBCD* operon variants were grown in LB to OD 0.5 and induced with 10 µM IPTG. After 3 h, bright field imaging was performed with the Nikon Ti-E inverted microscope using the Nikon Plan Apo ×100 oil objective, 1.40 NA and Photometrics CoolSNAP HQ2 CCD camera. Images were processed using the Nikon Elements 4.51 software.

### Purification of PylB variants

Protein purification was performed using a procedure adapted from the methods described by Quitterer et al.^22^. *E. coli* strain BL21 (DE3) cells containing either plasmids JH767, JH768v2, or JH769v2—which encode different *pylB* variants containing an N-terminal His-tag followed by a TEV protease cleavage site (see Supplementary Table 6)—were initially grown overnight in 20 mL of 2xYT media containing kanamycin at 37 °C. Cultures were subsequently diluted into 800 mL of 2xYT media and grown at 25 °C until *A*_600_ reached 0.4–0.6. Cells were then induced by adding 500 µM IPTG, and were grown overnight at 25 °C. Cultures were next pelleted, and resuspended in 25-40 mL of PylB Lysis Buffer, containing 100 mM Tris hydrochloride (pH 8.0), 500 mM NaCl, 5 mM sodium dithionite, and 15 mM imidazole hydrochloride. Cells were lysed by sonication, and then centrifuged at 17,000 × *g* for 1 h at 4 °C. Gravity columns containing Nickel-NTA resin were equilibrated with PylB Lysis Buffer, and clarified protein supernatants were applied to separate columns. Samples were eluted with PylB Elution Buffer, containing 100 mM Tris hydrochloride (pH 8.0), 500 mM NaCl, 5 mM sodium dithionite, and 500 mM imidazole hydrochloride. SDS-PAGE was used to confirm purification of His-tag containing PylB from each eluate and to determine protein purity (see Supplementary Fig. 12).

For each sample, buffer exchange into PylB Lysis buffer was subsequently performed using Amicon Ultra-15 centrifugal filtration units (3000 Da nominal molecular weight limit). Protein concentrations were determined by measuring absorbance at 280 nm, and samples were diluted to 0.4 mg/mL. Samples were next digested with TEV protease (provided by New England Biolabs, cat. # P8112S) by incubating at 30 °C for 2 h. Undigested PylB and residual protease were subsequently removed from each sample by reverse purification using Nickel-NTA resin. SDS-PAGE was subsequently used to determine the purity of the cleaved PylB samples. To prepare samples under low-salt conditions, aliquots of each sample were taken following purification samples and were dialyzed against low-salt lysis buffer, containing 100 mM Tris hydrochloride (pH 8.0), 20 mM NaCl, 5 mM sodium dithionite, and 15 mM imidazole hydrochloride.

### Quantification of PylB protein affinity purification yields

For quantitative yield assessment of PylB variants, purification of uncleaved His-tagged variants was performed as described above (without performing TEV protease digestion). Each variant was purified in biological triplicate, beginning from separate colonies. Purifications were performed using a 100 mL expression volume of 2xYT. Total protein concentrations were determined by measuring absorbance at 280 nm, and PylB purity was determined using SDS-PAGE.

### PylB solubility assays

Solubiilty assays were performed using a procedure adapted from Kramer et al.^29^, using cleaved PylB samples (without His tags) purified as described above. Each protein sample was initially concentrated to a uniform concentration of 2.6 mg/mL. Separate aliquots of each protein sample were next mixed with solutions of either ammonium sulfate or PEG-8000 in a one-to-one ratio. For ammonium sulfate, solutions were prepared at concentrations ranging from 0.5 to 1.5 M; for PEG-8000, solutions were prepared at concentrations ranging from 5 to 15% (w/v). After thorough mixing, the solutions were allowed to equilibrate at room temperature for 10 min. Solutions were subsequently centrifuged at 17,000 × *g* for 10 min, and the resulting supernatant was transferred into fresh tube. Concentration of each supernatant was determined by measuring absorbance at 280 nm. This protein concentration corresponds to the solubility of each protein at the corresponding concentration of precipitant.

### Analysis of PylB solubility

Protein solubility data collected at different precipitant concentrations were subsequently analyzed. The following formula^29^ was used to describe the relationship between precipitant concentration and protein solubility:

1 $${\mathrm{Log}}\,{{S}}=\,{\mathrm{Log}}\,{{{S}}}_{0}-\beta\; [{\rm{precipitant}}]$$

In this expression, S is the measured solubility at a given concentration of precipitant, S_0_ is the solubility in the absence of precipitant, and *β* is the solubility constant, denoting the dependence of solubility on precipitant concentration for a given protein. Log protein concentration measurements were plotted against precipitant concentrations, and *β* values as well as uncertainties were determined for each PylB variant using linear regression function in Microsoft Excel. Points lying outside the linear region of the plot were excluded from the regression analysis. Here we define aggregation propensity as negative *β* values (−*β*).

### Biosynthetic production and extraction of Pyl

To evaluate Pyl production, the pylBCD pathway was expressed inside *E. coli* cells and free Pyl was extracted^4,35^. *E. coli* strain BL21(DE3) cells were transformed with either plasmid JH572 (encoding the empty pBAD vector), JH577 (encoding *pylBCD* variant 3f2), or plasmid JH579 (encoding variant JM10.1). Cells were inoculated into 100–500 mL of LB broth supplemented with 1 mM l-lysine, and grown at 37 °C. Upon reaching *A*_600_ = 0.3, cultures were induced with 1.3 mM l-arabinose, and subsequently grown at 37 °C for periods ranging from 4 to 18 h. Cells were subsequently pelleted and washed with distilled water. Cells were subsequently extracted with either 2 mL of methanol per gram of wet cell weight at room temperature, or with 2 mL of 66% aqueous methanol at 70 °C. Extractions were incubated for 30 min at the tested temperatures. Extracts were then centrifuged at 13,000 × *g* for 5 min, and the supernatant was retained. For a subset of samples an additional filtration step was then performed, wherein samples were passed through an Amicon Ultra-15 Centrifugal Filter unit (3000 NMWL), retaining the flow-through; samples were diluted with distilled water to 60% methanol prior to filtration. For another subset of samples an ethyl acetate extraction was then performed, wherein ethyl acetate was added to each sample until reaching a total concentration of 80%; samples were then centrifuged at 13,000 × *g* for 5 min, and the supernatant was retained. Extracts were dried during centrifugation under vacuum prior to analysis.

### DSF assays of PylB variants

Protein DSF assays (also known as the thermofluor assay) were used to evaluate thermostability of protein samples^31^. Briefly, 50 µL reaction mixtures were prepared in white 96-well PCR plates (Bio-Rad Laboratories, cat. # HSP9655), with each mixture containing purified PylB samples diluted to 5 µM as well as SYPRO orange dye (Sigma-Aldrich cat. # S5692) at 20× working concentration. Sealed plates were run on a Bio-Rad CFX Connect Real-Time PCR machine, heating from 25 to 95 °C. Samples were heated at a rate of 0.5 °C per 30 s, and fluorescent readings were taken at every 30 s interval. All protein samples were run in triplicate, including separate low salt and high salt negative controls which contained only buffer and dye (no protein). To plot melting curves, averages of each protein sample were calculated and the background signal from the negative controls was subtracted. Melting temperature (*T*_m_) values were identified by determining the temperature at which the first derivative of the fluorescent signal reached its lowest minimum value. For experiments containing SAM or l-lysine additives, these compounds were each added to a final working concentration of 10 mM. Given the high lability of SAM, experiments using this compound were performed within the same day of the arrival of this reagent (Sigma-Aldrich cat. # A7007).

### Thin-layer chromatography analysis of Pyl extracts

Thin-layer chromatography (TLC) was used for separation and quantification of amino acids in cell extracts. Dried extract samples were resuspended in methanol and spotted onto Cellulose 400 TLC plates (100 µM, 20 × 20 cm). Quantity of extracts spotted were empirically optimized by trial and error. Plates were run twice in the same dimension using a 1-butanol/acetone/glacial acetic acid/water (35/35/7/23%) mobile phase. Plates were dried for 1 h after running. To visualize amino acids, plates were uniformly sprayed with ninhydrin (0.125% w/v, dissolved in acetone) and developed by incubating at 70 °C for 5–10 min. Developed samples were visualized under white light.

### Cellular fluorescence analysis of Pyl extracts

*E. coli* strain BL21 (DE3) and strain C321.∆A.exp cells were separately transformed with plasmid JH528, encoding arabinose-inducible sfGFP with a single amber codon as well as constitutively expressed *pylS* and *pylT* (see Supplementary Table 6). Separately, dried extract samples were resuspended in distilled water. Cells cultures were grown to *A*_600_ 0.2–0.5 in LB media, and subsequently mixed with an equal volume of resuspended extract. Cultures were simultaneously induced with 1.3 mM l-arabinose, and grown for 3–18 h. Fluorescence measurements were performed during cell growth, as described in the “Fluorescence-based *pylBCD* activity assay” section above.

### PylB proteolytic degradation assays

Solid α-chymotrypsin (Sigma-Aldrich, cat. # C3142) stock solution was first prepared by dissolving the enzyme in 1 mM HCl to a concentration of 0.05 mg/mL; chymotrypsin solutions were stored for up to 5 days following resuspension. Purified and His-tag-cleaved PylB samples were each concentrated to a uniform concentration of 1 mg/mL,120 µL aliquots of each sample were added to a separate tube, supplemented with 10 mM CaCl_2_, and allowed to warm to room temperature. Tubes were then pre-labeled for each time point for each sample, and 6.7 µL SDS-PAGE loading dye was added to each tube. Prior to initiating digestion, an undigested 24 µL aliquot of each sample was removed and mixed with loading dye and 2.8 µL of 1 mM HCl (termed the 0-min sample). To the remaining 96 µL of each protein sample, 9.6 µL of chymotrypsin solution was then added, and post-digestion time was recorded. Aliquots were taken from each sample at the following time-points: 30 s, 1 min, 2 min, 5 min, 10 min, and 15 min. Upon being removed from the digestion reaction, each aliquot was mixed with loading dye in a separate tube, and immediately placed into a heat-block preheated to 98 °C. Each sample was boiled for 15 min, chilled on ice for at least 2 min, and a 4-8 µL aliquot was then run on a pre-cast SDS-PAGE gel (Bio-Rad cat. #4568096) before staining with Coomassie blue dye. As a standard, 4 µL of BLUEstain 2 protein ladder (GOLDBIO cat. #P008-500) was also loaded on each gel. Stained gels were imaged, and PylB bands were quantified in each sample using ImageJ; band intensities were converted into protein concentration based on observed intensities of ladder samples, as the concentration of the ladder was provided by the supplier. At each time point, the amount of enzyme product (degraded PylB) produced was calculated by subtracting the PylB concentration at that time point from the initial PylB concentration. From these data, catalytic constant (*k*_cat_) values for chymotrypsin degradation of each PylB variant were calculated using Graphpad Prism 8.

### Quantitative PylB western blot analysis

Clarified lysates were initially prepared for immunoblot analysis using *E. coli* BL21(DE3) cells transformed with expression constructs for different PylB variants (see Supplementary Table 9 and “Purification of PylB variants” section). Isolated colonies were first grown overnight in 3 mL of LB broth containing kanamycin at 37 °C. Cultures were subsequently back-diluted into flasks containing 100 mL of 2xYT media with kanamycin, and were grown at 25 °C until *A*_600_ values reached 0.4–0.6. Cells were then induced by adding 500 µM IPTG, and were grown overnight at 25 °C. Following overnight growth, *A*_600_ measurements were taken for all samples, and cultures were serially diluted and plated to determine the colony-forming units (CFUs/mL) for each sample. Cultures were next pelleted, and resuspended in 10 mL of PylB Lysis Buffer, containing 100 mM Tris hydrochloride (pH 8.0), 500 mM NaCl, 5 mM sodium dithionite, and 15 mM imidazole hydrochloride. Cells were lysed by sonication, and then centrifuged at 17,000 × *g* for 1 h at 4 °C, retaining the clarified supernatant.

Lysate samples were normalized by protein concentration (Thermo Scientific NanoDrop Lite) through dilution in sample buffer (500 mM Tris pH 8.0, 4% (w/v) lithium dodecyl sulfate, 1 mM EDTA, 20% (w/v) glycerol, 0.44 mM Coomassie blue G250, 0.332 mM phenol red, 50 μM DTT) and heated at 100 °C for 5 min. Equal volumes of samples were loaded alongside pre-stained markers (New England Biolabs, P7712S) into Bolt 10% (w/v) bis-tris gels (Invitrogen) and electrophoresed in MOPS buffer (50 mM MOPS free acid, 50 mM Tris base, 0.1% (w/v) sodium dodecyl sulfate, 1 mM EDTA). Proteins were transferred from the gel to Hybond-ECL nitrocellulose membranes (Amersham, Protran Premium 0.45 μm NC 10600003) using a GenScript eBlot L1 transfer system and standard transfer settings. The membranes were air-dried for 1 h followed by 1 h blocking with Intercept® Blocking Buffer (LI-COR) at 4 °C with rocking.

Membranes were incubated overnight at 4 °C with rocking with mouse primary antibodies diluted in 8% non-fat dry milk in Tris-buffered saline with Tween-20 (TBST; 20 mM Tris pH 7.5, 150 mM NaCl, 0.1% (v/v) Tween-20). Primary antibodies were raised against GAPDH (Invitrogen MA5-15738 1:5000 for replicates 1–3 or 1:200 for replicates 4–6) or 6x-His-Tag (ThermoFisher MA121315 1:250 for replicates 1–3 and 1:10,000 for replicates 4–6). After rinsing with TBST, membranes were incubated at 4 °C with rocking for 2–12 h with IRDye 680RD-linked goat anti-mouse secondary antibodies (LI-COR 926-68070 diluted 1:10,000 in TBST with 8% non-fat dry milk for replicates 1–3 and 1:2500 for replicates 4–6). Secondary antibodies were visualized using a LI-COR Odyssey FC imaging system. Membranes were probed with primary antibodies sequentially, without stripping. Quantification was performed using Image Studio software (LI-COR, version 5.2).

### Reporting summary

Further information on research design is available in the Nature Research Reporting Summary linked to this article.

## Supplementary information

Supplementary Information

Supplementary Data 1

Description of Additional Supplementary Files

Reporting Summary

## Data Availability

Data supporting the findings of this work are available within the paper and its Supplementary Information files. A reporting summary for this Article is available as a Supplementary Information file. DNA sequence data originating from *M. acetivorans* are publicly available under Genbank accession number AE010299. Source data are provided with this paper.

## Source data

Source Data
