# Supplementary material for: Improved pyrrolysine biosynthesis through phage assisted non-continuous directed evolution of the complete pathway
Source: Nat Commun. 2021 Jun 24;12:3914. doi: 10.1038/s41467-021-24183-9 (PMC8225853; doi:10.1038/s41467-021-24183-9)
Supplement: Supplementary file 1 — Supplementary Information [file 41467_2021_24183_MOESM1_ESM.pdf]

Improved pyrrolysine biosynthesis through phage assisted non-continuous directed evolution of the complete pathway

Ho *et al.*

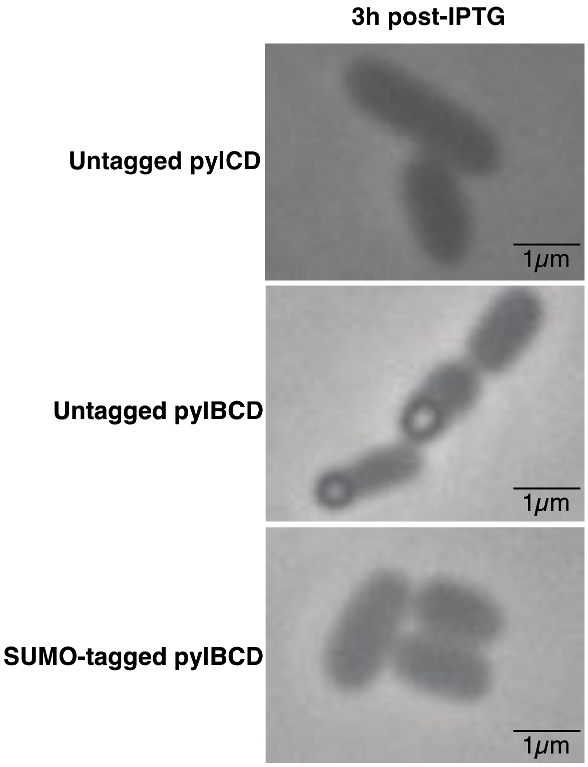

Supplementary Figure 1. Microscopy of *E. coli* cells expressing *pylBCD*. Cells expressing WT *M. acetivorans* genes *pylC* and *pylD* from plasmid JH60 are shown in upper panel; cells expressing the WT *M. acetivorans pylBCD* pathway from plasmid JH123 are shown in the middle panel; cells expressing *pylBCD* following the addition of a SUMO tag to the N-terminus of *pylB* (SUMO-*pylBCD*) from plasmid JH126 are shown in lower panel. While inclusion bodies are visible in cells expressing the WT *pylBCD* pathway, they are not observed in the absence of *pylB* or in cells expressing SUMO-*pylBCD*. For each strain shown above, microscopy experiments were repeated across three independent replicates yielding similar results.

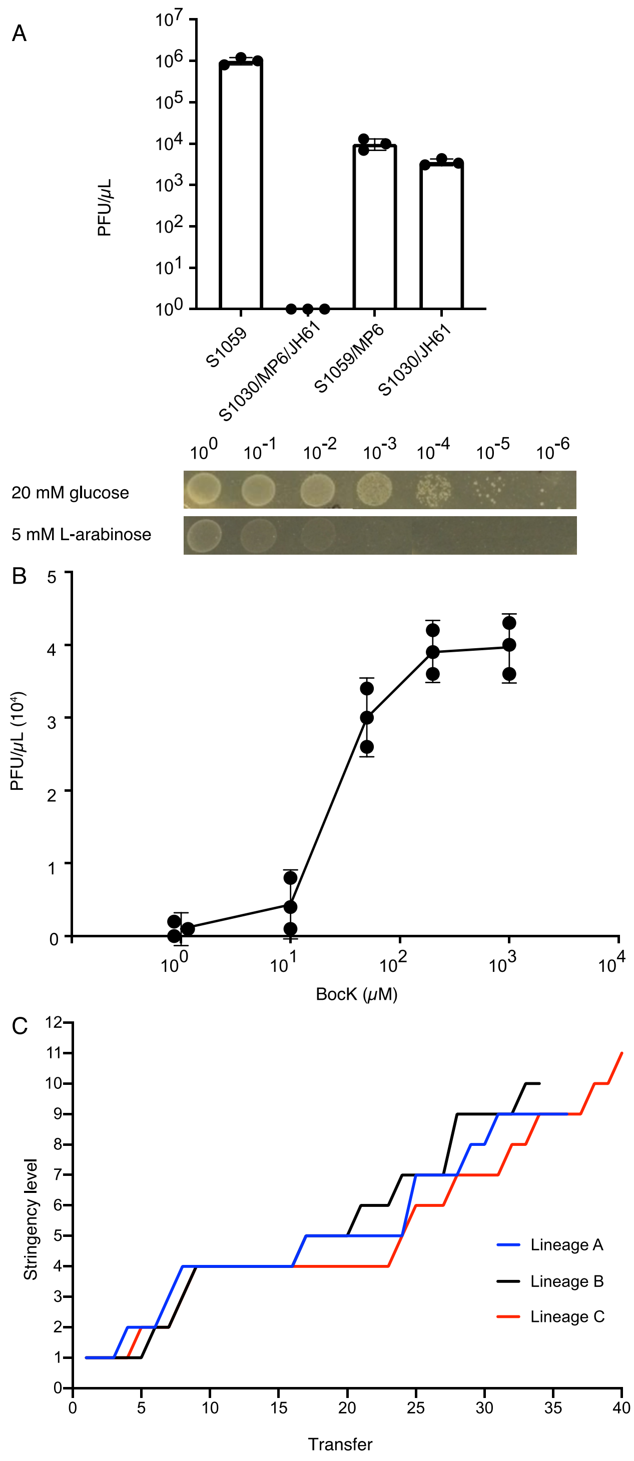

Supplementary Figure 2. Alt-PANCE of *pylBCD* evolution details. (A) Phage titers are shown (in PFU/µL) for ancestral SP.BCD using different *E. coli* host cells. Compared to the permissive growth conditions within strain S1059, phage titers are reduced when subjected to mutagenesis (in strain S1059/MP6) or selection (in strain S1030/JH61). When simultenously subjected to both selection and mutagenesis (in strain S1030/MP6/JH61), no phage growth is observed. Error bars are derived from samples tested across three independent biological replicates; data shown represents mean values ± s.d. (B) Glucose-repression and arabinose-activation of mutagenesis in PANCE host cells. Error bars are derived from samples tested across three independent technical replicates; data shown represents mean values ± s.d. (C) BocK supplementation curve—Alt-PANCE was initiated at the BocK concentration that provided 80% maximum PFU/µL. (D) Stringency ramping curve for

S1030/AP, corresponding to increasing stringency levels 1–11 over the course of 34–40 rounds of Alt-PANCE.

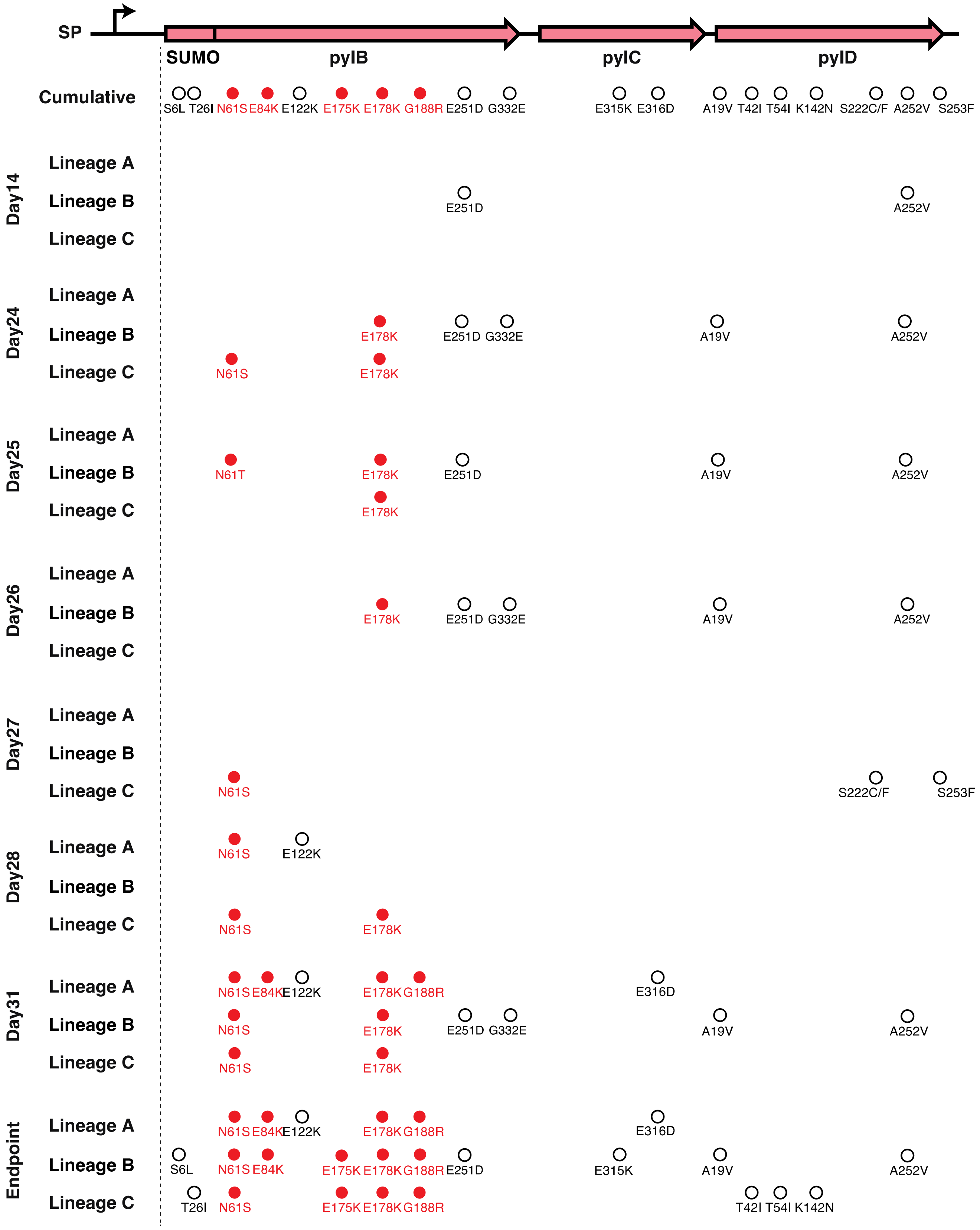

Supplementary Figure 3. Evolutionary timeline. Mutations shown were identified at different time points during Alt-PANCE of *pylBCD*. Mutations were identified by sequencing isolated SP.BCD plaques during evolution. Convergent mutations are shown in red.

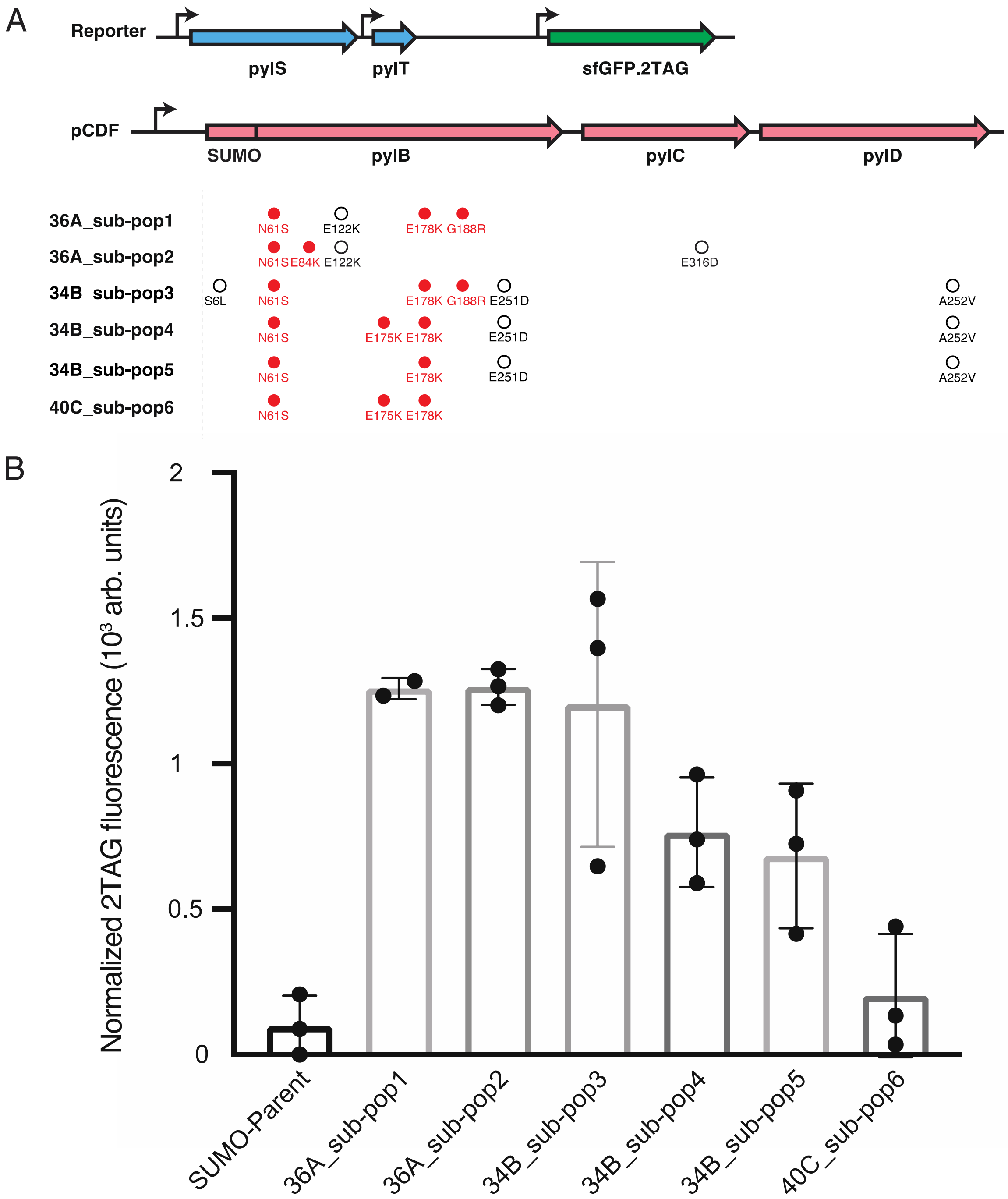

Supplementary Figure 4. Fluorescence-based plate reader assay with six evolved variants. (A) Operon maps are shown for the reporter plasmid 2VecJH.Cam.sfGFP.2TAG and the pCDF expression vector containing *pylBCD* variants (see Methods). Mutations within each variant tested are shown to the right of each variant name. Each mutation is shown below the corresponding gene in which it is found (e.g., *pylB*, *pylC*, or *pylD*). (B) Normalized fluorescence following induction in *E. coli* strain BL21 (DE3) cells containing the plasmids described above (see Methods). Fluoresence mediated by the ancestral variant (SUMO-Parent) is shown on the far left, while fluoresence mediated by various evolved *pylBCD* variants are shown to the right. Error bars shown reflect standard deviation, and are derived from testing three independent biological replicates; data shown represents mean values ± s.d.

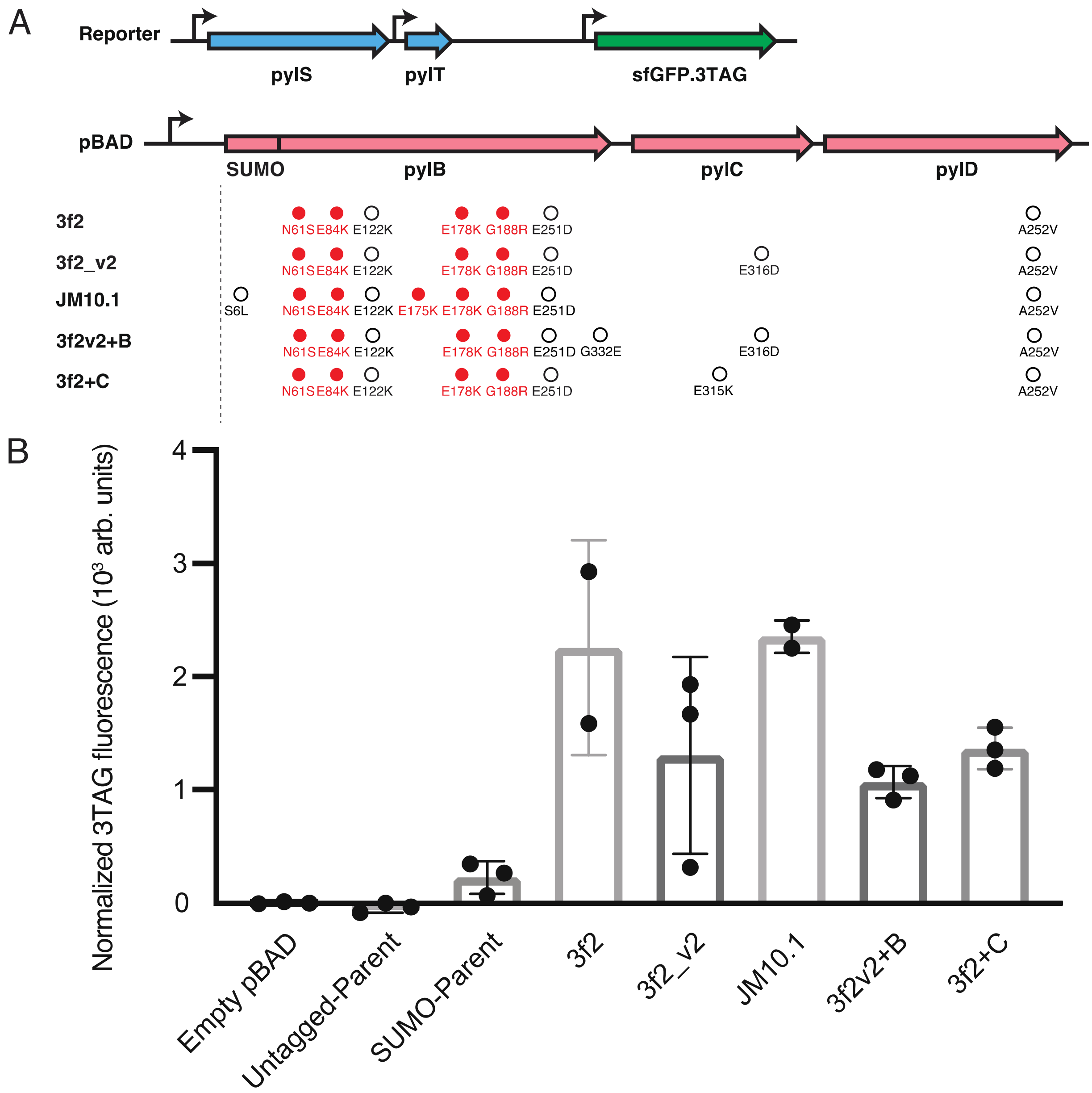

Supplementary Figure 5. Fluorescence-based plate reader assay with five combinatorial variants. (A) Operon maps are shown for the reporter plasmid 2VecJH.Cam.sfGFP.3TAG and the pBAD expression vector containing *pylBCD* variants (see Methods). Mutations within each variant tested are shown to the right of each variant name. Each mutation is shown below the corresponding gene in which it is found (e.g., *pylB*, *pylC*, or *pylD*). (B) Normalized fluorescence following induction in *E. coli* strain C321.ΔA.exp cells containing the plasmids described above (see Methods). Fluoresence mediated by the empty pBAD vector, the WT *pylBCD* pathway (termed 'untagged parent'), and the SUMO-tagged ancestral variant (SUMO-Parent) are shown on the left, while fluoresence mediated by various evolved *pylBCD* variants are shown to the left. Error bars shown reflect standard deviation, and are derived from testing three independent biological replicates; data shown represents mean values ± s.d.

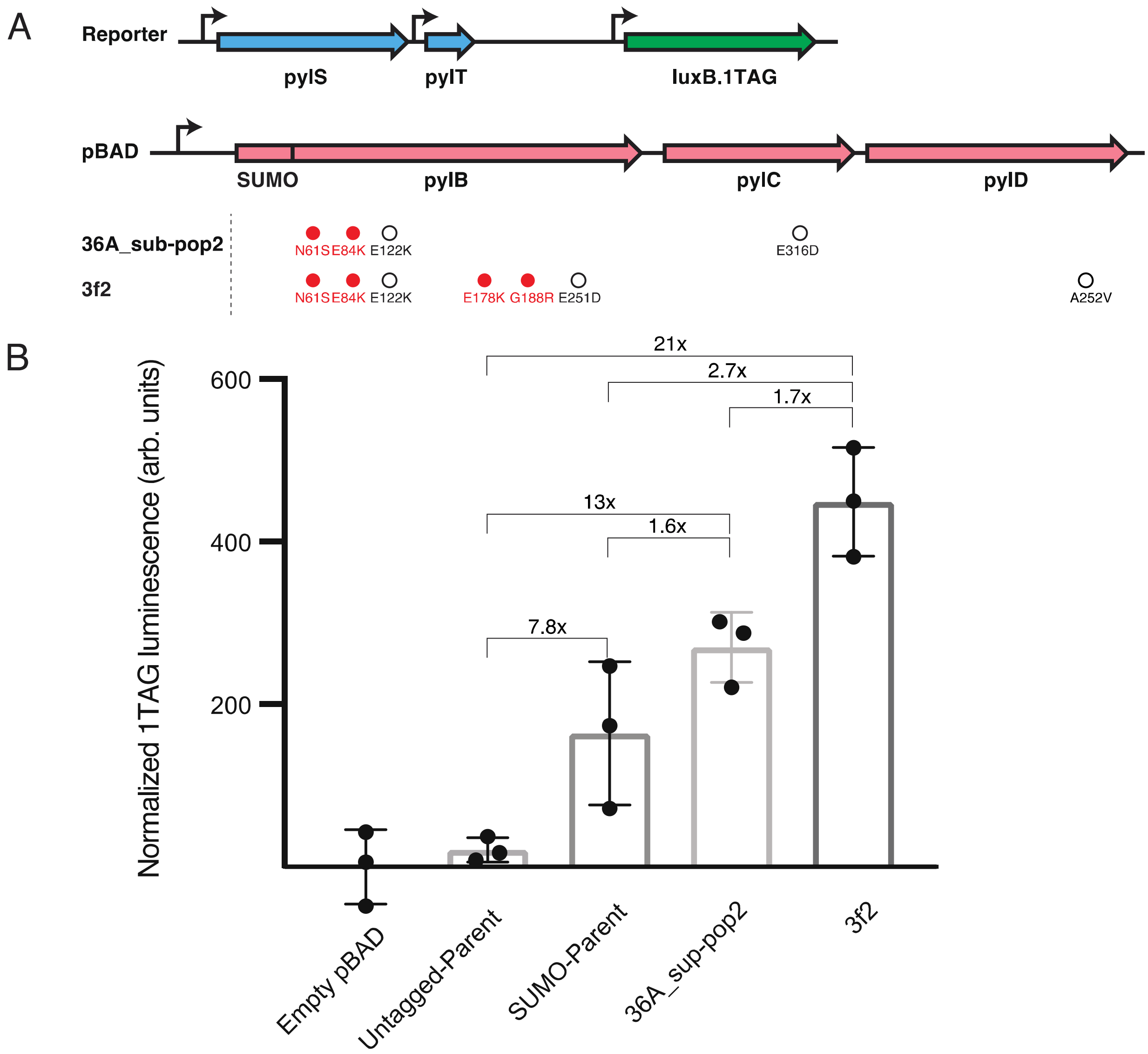

Supplementary Figure 6. Luminescence-based plate reader assay with the evolved and combinatorial variants. (A) Operon maps are shown for the reporter plasmid 2VecJH.Lux.1TAG and the pBAD expression vector containing *pylBCD* variants (see Methods). Mutations within each variant tested are shown to the right of each variant name. Each mutation is shown below the corresponding gene in which it is found (e.g., *pylB*, *pylC*, or *pylD*). (B) Normalized luminescence following induction in *E. coli* strain C321.ΔA.exp cells containing the plasmids described above (see Methods). Luminescence mediated by the empty pBAD vector, the WT *pylBCD* pathway (termed 'untagged parent'), and the SUMO-tagged ancestral variant (SUMO-Parent) are shown on the left, while luminescence mediated by various evolved *pylBCD* variants are shown to the left. Luminescence results reveal the increasing biosynthetic yield of the pathway after the Sumo tag is appended to *pylB* (7.8-fold), after PANCE evolution (13-fold), and after screening different combinations of the emergent mutations (21-fold). Error bars shown reflect standard deviation, and are derived from testing three independent biological replicates; data shown represents mean values ± s.d.

AA: F. S V R G E G E G D A T O G K L .T (N+123)
DNA: AGC GTG CGC GGC GAA GGT GAA GGC GAT GCA ACC TAG GGT AAA CTG

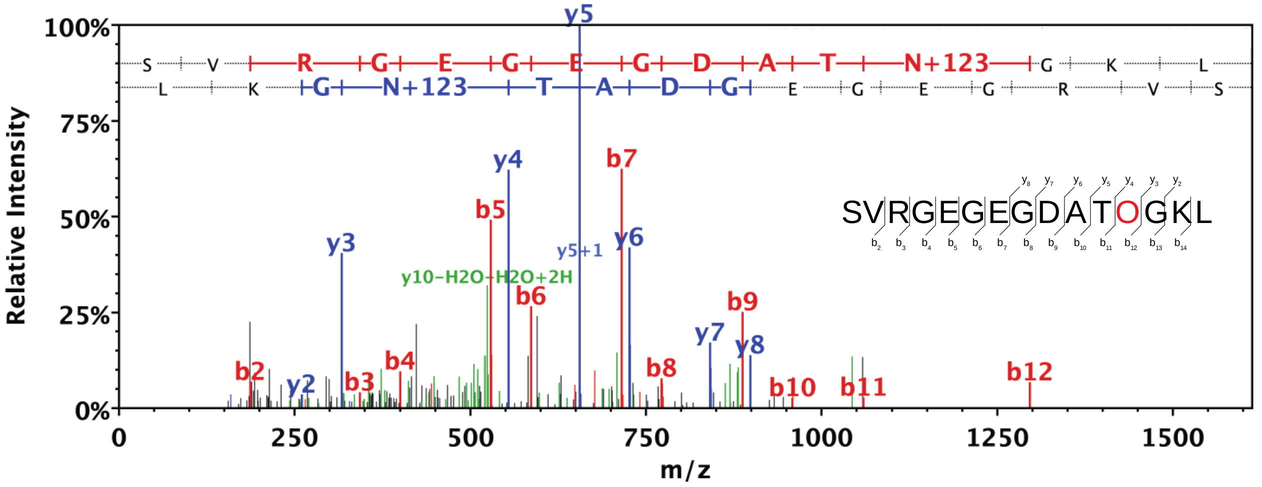

Supplementary Figure 7. LC-MS/MS spectrum of Pyl-sfGFP confirming Pyl insertion at position N39O. We performed searches for molecular mass shifts indicative of Pyl incorporation (+123.19 g/mol for N to O) and potential desmethyl-pyrrolysine (dmPyl) incorporation (+109.16 g/mol for N to dmPyl). Prior work by Gaston *et al* (2011)[1] showed that cells expressing PylC and PylD (lacking PylB) grown in media supplemented with D-ornithine will produce a side product, dmPyl. LC-MS/MS did not detect any dmPyl incorporation, which was expected since dmPyl requires supplementation of media with D-ornithine and was not performed here. This observation indicates that the evolved pathway produced only Pyl, as intended and expected. Detailed fragmentation information in Supplementary Table 4.

AA: Y. N F N S H N V O I T A D K Q K N G I K A N F.K (Y+74)
DNA: AAC TTC AAT TCG CAC AAC GTG TAG ATC ACC GCA GAT AAG CAG AAG AAC GGT ATC AAG GCT AAC TTC

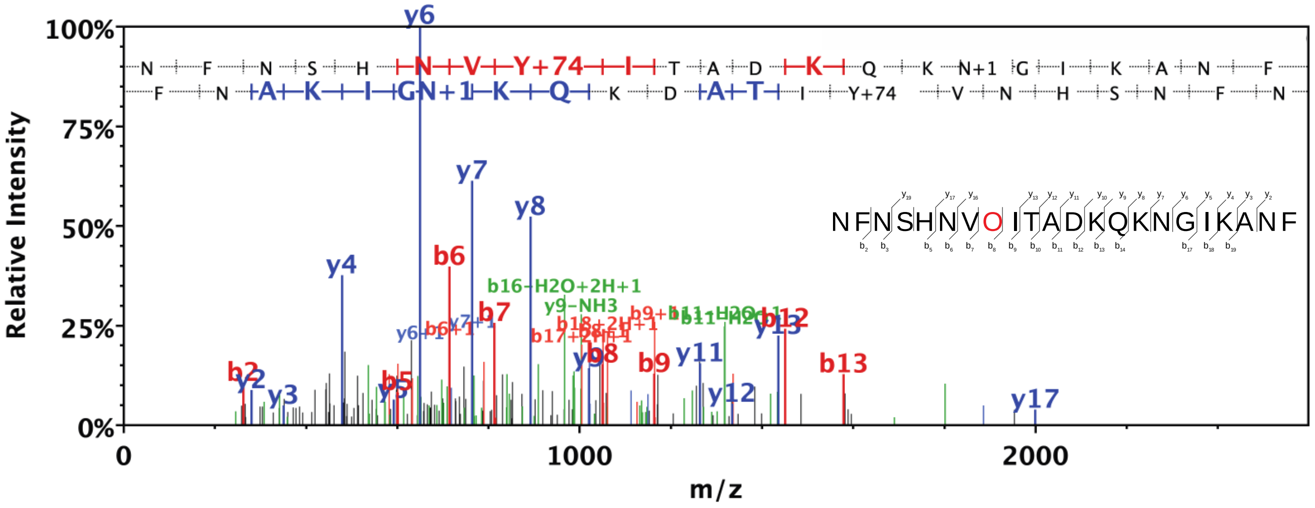

Supplementary Figure 8. LC-MS/MS spectrum of Pyl-sfGFP confirming Pyl insertion at Y151O. We performed searches for molecular mass shifts indicative of Pyl incorporation (+74.12 g/mol for Y to O) and potential dmPyl incorporation (+60.09 g/mol for Y to dmPyl). LC-MS/MS did not detect any dmPyl incorporation, which was expected since dmPyl requires supplementation of media with D-ornithine and was not performed here. This observation indicates that the evolved pathway produced only Pyl, as intended and expected. Detailed fragmentation information in Supplementary Table 5.

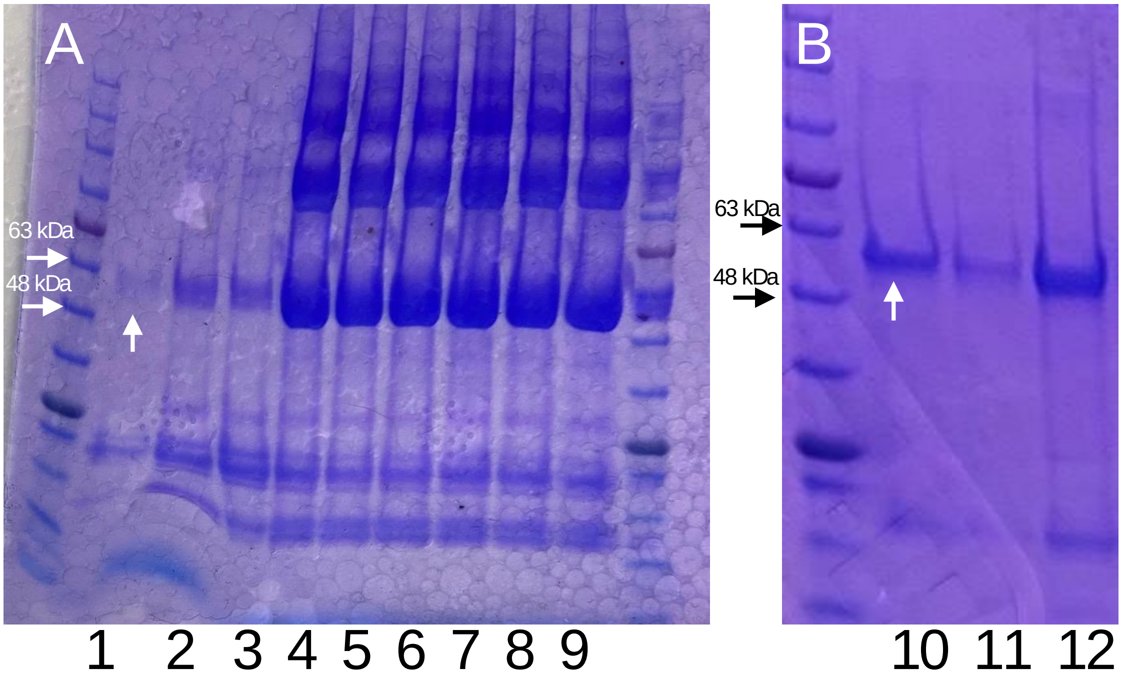

Supplementary Figure 9. SDS-PAGE analysis of purified PylB samples. (A) Three PylB mutants containing N-terminal histidine tags were each purified in triplicate under similar conditions (see Methods). Labeled samples 1-3 correspond to variant SUMO-PylB; samples 4-6 correspond to variant PylB.3f2 samples; Samples 7-9 correspond to variant PylB.JM10.1 samples. PylB was estimated at ~37% purity for each sample containing histidine tags. (B) Histidine tags were cleaves from each purified protein, and samples were further purified by reverse Ni-NTA chromatography. Sample 10 corresponds to pooled variant SUMO-PylB samples; sample 11 corresponds to pooled variant PylB.3f2 samples; sample 12 corresponds to pooled variant PylB.JM10.1 samples. PylB was estimated at ~90% purity for each sample following cleavage of histidine tags. White arrows indicates the expected position of PylB (~52 kDa) on each gel. BLUEstain™ 2 Protein ladder (GoldBio) was run alongside samples on each gel; molecular weight markers lying above and below PylB (63 kDa and 48 kDa, respectively) are labeled on each gel. For each PylB variant in panels A and B, protein purifications were repeated across three independent replicates yielding similar results.

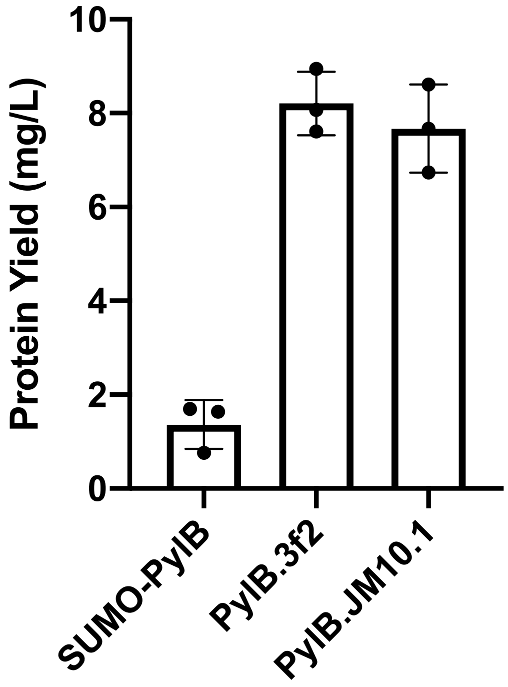

Supplementary Figure 10. Protein yields of PylB variants. Protein variants were overexpressed in *E. coli* strain BL21 (DE3) cells following IPTG induction (see Methods). Yields of His-tag containing proteins purified by metal affinity chromatography are shown in mg of PylB protein produced per liter of cell culture, adjusted for protein purity. PylB variants 3f2 and JM10.1 produced 6.0-fold and 5.6-fold greater protein yields, respectively, compared to SUMO-PylB. Protein samples were purified from biological triplicate cultures; individual values are shown, with the standard deviation shown as uncertainty. Averaged (mean) values are shown as the measure of center for each error bar.

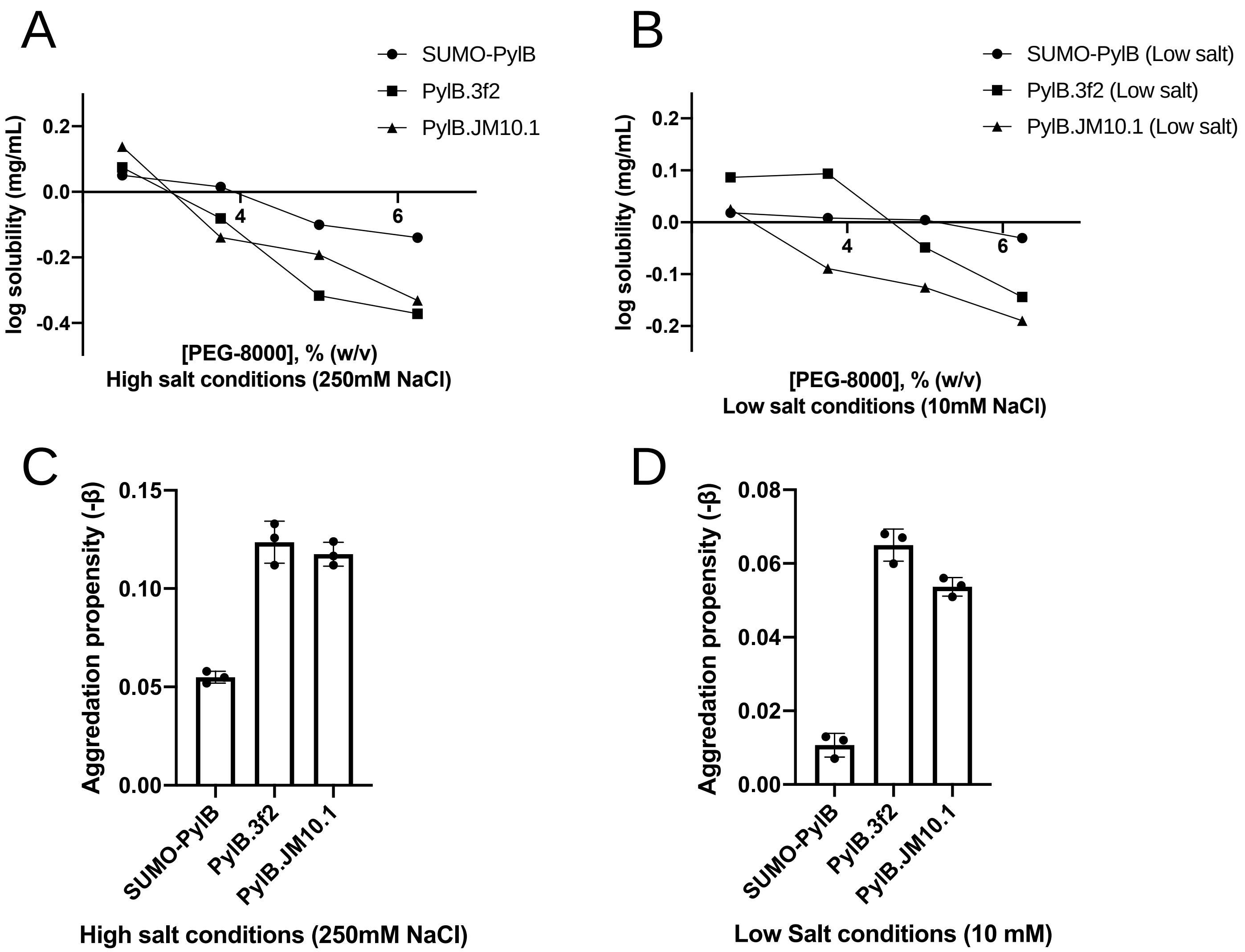

Supplementary Figure 11. PylB variant solubility assays. Purified and His-tag cleaved protein samples of PylB variants were exposed to varying concentrations of the precipitant PEG-8000, and maximum protein solubility for each sample was plotted against the corresponding precipitant concentration (see Methods). Protein samples used were prepared at ≥90% purity. Solubility assays were separately conducted under (A) high salt conditions (250 mM NaCl) and (B) low salt conditions (10 mM NaCl). Aggregation propensity (-β) is shown for each PylB protein variant following both (C) high salt assays and (D) low salt assays. Higher aggregation propensity indicates reduced protein solubility. Aggregation propensity (-β) values were calculated for each PylB variant using linear regression in Microsoft Excel (see Methods). Data collected from samples tested against four concentrations of PEG-8,000 were included in the analysis. Error bars for (C) and (D) represent standard deviation, and are derived from samples tested across three independent technical replicates. Source data underlying Supplementrary Figures 11A and 11B are provided as a Source Data file.

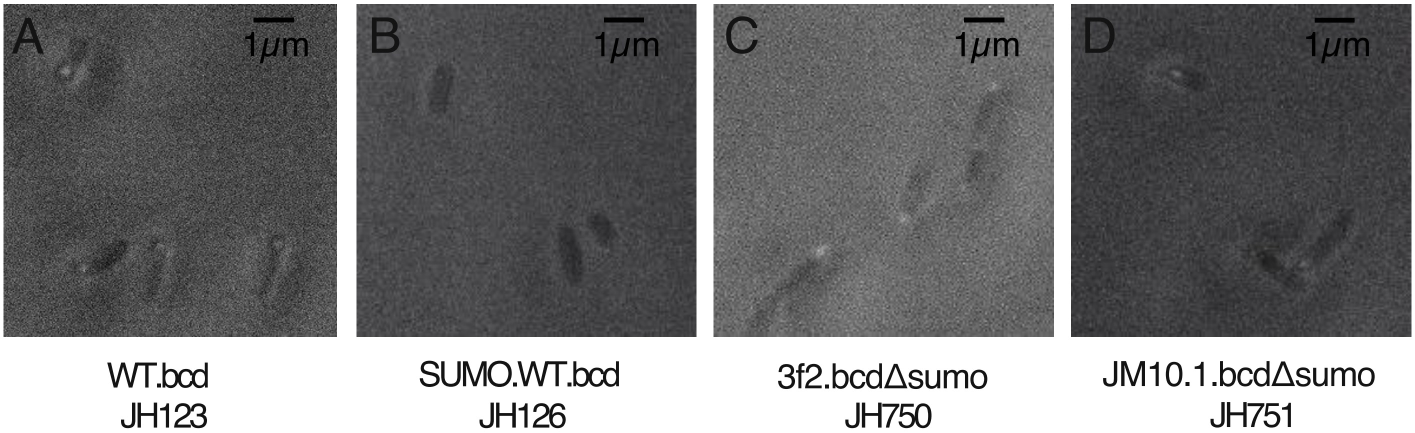

Supplementary Figure 12. Microscopy of *E. coli* cells expressing mutant *pylBCD* variants. Cells expressing different variants of the *pylBCD* pathway. The corresponding plasmid name expressing each variant is shown below each image. Cells shown are expressing (A) WT *M. acetivorans pylBCD*, (B) SUMO-*pylBCD*, (C) evolved variant 3f2 with the SUMO tag removed, and (D) evolved variant JM10.1 with the SUMO tag removed. Inclusion bodies can be observed in all samples without SUMO tags (panels A, C, and D) but are not observed in panel B. For each strain shown above, microscopy experiments were repeated across three independent replicates yielding similar results.

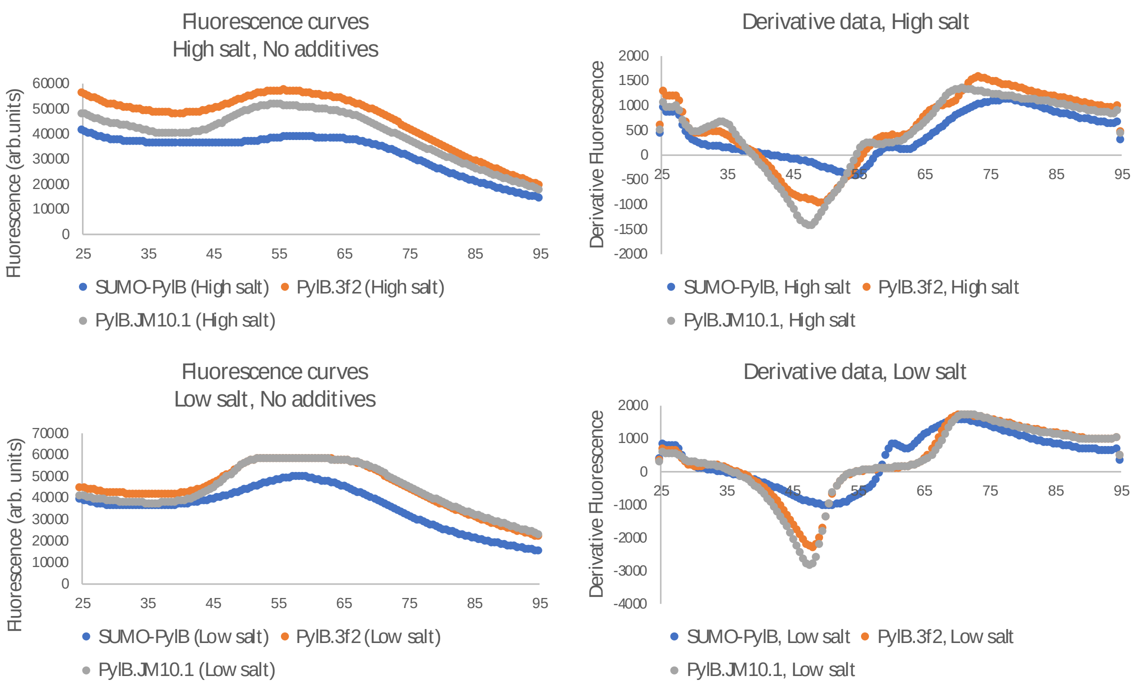

Supplementary Figure 13. Differential scanning fluorimetry (DSF) assays of PylB variants under high and low salt. Purified PylB samples were gradually heated, and a fluorescent probe was used to evaluate the extent of protein denaturation at each temperature (see Methods). Fluorescence values are shown in the curves on the left, while derivative fluorescence is shown in the curves on the right. Curves shown are averaged across 3 replicates; background subtraction was also performed using data collected using negative control samples that did not contain protein (see Methods). Samples were tested under both low salt (10 mM) and high salt (500 mM) conditions. Across both salt conditions, the ancestral SUMO-PylB variant showed a less pronounced transition but a higher Tm value compared to the evolved variants. Source data are provided as a Source Data file.

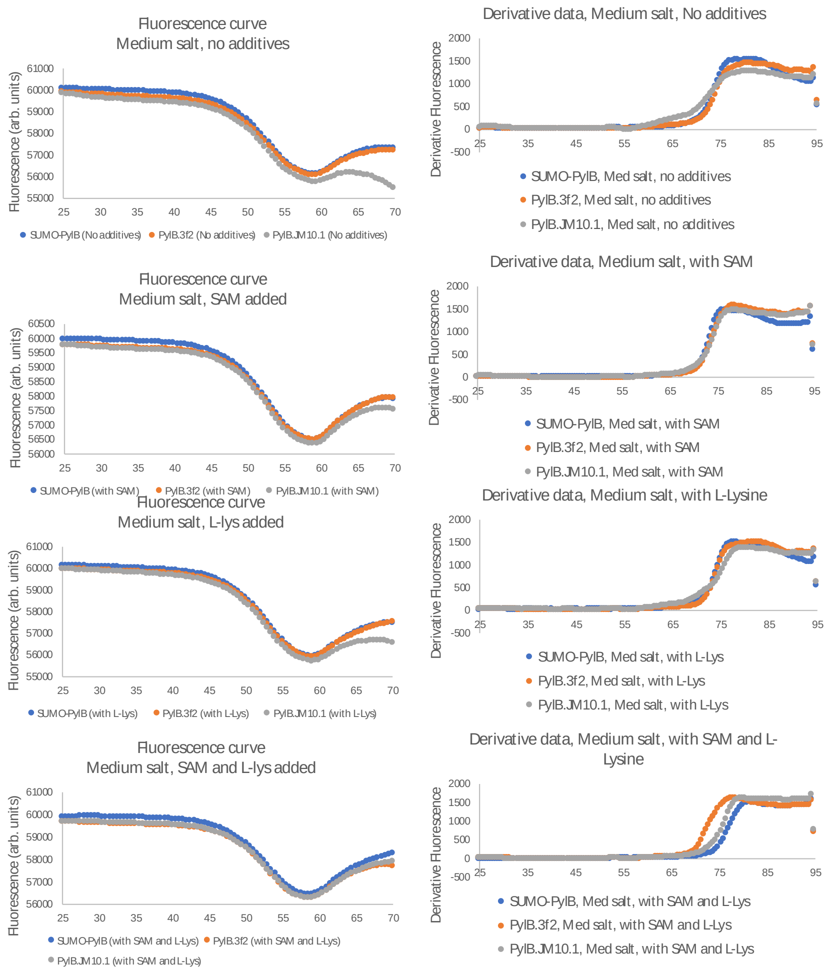

Supplementary Figure 14. Differential scanning fluorimetry (DSF) assays of PylB variants supplemented with SAM and/or L-lysine. Purified PylB samples were gradually heated, and a fluorescent probe was used to evaluate the extent of protein denaturation at each temperature (see Methods). Fluorescence values are shown in the curves on the left, while derivative fluorescence is shown in the curves on the right. Curves shown are averaged across 3 replicates; background subtraction was also performed using data collected using negative control samples that did not contain protein (see Methods). Assays were performed under medium salt (350 mM) conditions. Samples were tested without any additional additives, with the addition of SAM (10 mM), with the addition of L-lys (10 mM), and with the addition of both SAM and L-Lys. Across each condition tested, ancestral and evolved PylB variants showed similar transitions to one another. Source data are provided as a Source Data file.

Supplementary Table 1. Evolutionary selection conditions.

Level	[BocK]/ μM	PylRS variant	Genotype of *gIII*	PylRS level	Plasmid No.
**1**	200	Chimeric PylRS	S12O	High	JH61
**2**	50	Chimeric PylRS	S12O	High	JH61
**3**	25	Chimeric PylRS	S12O	High	JH61
**4**	10	Chimeric PylRS	S12O	High	JH61
**5**	0	Chimeric PylRS	S12O	High	JH61
**6**	0	Chimeric PylRS	S12O	Low	JH19
**7**	0	Chimeric PylRS	S12O, Y166O	Low	JH76
**8**	0	Chimeric PylRS	S12O, P83O, Y166O	Low	JH77
**9**	0	32A-N-terminal	S12O	High	JH190
**10**	0	32A-N-terminal	S12O, Y166O	High	JH191
**11**	0	32A	S12O	Low	JH202

Selection stringency was gradually increased by withdrawal of BocK, use of PylRS variants with lower affinities for Pyl, incorporation of additional stop codons in gIII, and lowering the expression level of PylRS. Chimeric PylRS was previously prepared by fusion of the *Methanosarcina barkeri* PylRS N-terminal and *M. mazei* PylRS C-terminal domains, as previously reported[2]. Variant 32A and variant 32A-N-terminal are chimeric PylRS mutants with reduced recognition of Pyl[3].

Supplementary Table 2. List of mutations in the phage plaques sequenced at the endpoints of each lineage (A–C).

	SUMO		PylB							PylC		PylD				
	S6L	T26I	N61S	E84K	E122K	E175K	E178K	G188R	E251D	E315K	E316D	A19V	T42I	T54I	K142N	A252V
Line A plaque 1			N61S	E84K	E122K											
Line A plaque 2			N61S		E122K		E178K	G188R								
Line A plaque 3			N61S	E84K	E122K											
Line A plaque 4			N61S	E84K	E122K											
Line A plaque 5			N61S	E84K	E122K											
Line A plaque 10			N61S	E84K	E122K											
Line A plaque 11			N61S	E84K	E122K						E316D					
Line A plaque 12			N61S	E84K	E122K						E316D					
Line A plaque 13			N61S	E84K	E122K						E316D					
Line A plaque 15			N61S	E84K	E122K											
Line B plaque 2	S6L		N61S				E178K	G188R	E251D							A252V
Line B plaque 3			N61S				E178K		E251D							A252V
Line B plaque 4			N61S			E175K	E178K		E251D							A252V
Line B plaque 5			N61S					G188R	E251D							A252V
Line B plaque 6			N61S				E178K									A252V
Line B plaque 7			N61S				E178K		E251D							A252V
Line B plaque 8	S6L		N61S						E251D							A252V
Line B plaque 9							E178K			E315K						A252V
Line B plaque 10				E84K			E178K		E251D							A252V
Line B plaque 11			N61T					G188R	E251D			A19V				A252V
Line B plaque 14			N61S					G188R								A252V
Line B plaque 15			N61S				E178K		E251D							A252V
Line C plaque 1			N61S			E175K	E178K									
Line C plaque 2			N61S			E175K	E178K							T54I	K142N	
Line C plaque 3			N61S			E175K	E178K									
Line C plaque 4			N61S				E178K	G188R								
Line C plaque 5		T26I	N61S				E178K	G188R								
Line C plaque 6			N61S			E175K	E178K									
Line C plaque 7			N61S			E175K	E178K									
Line C plaque 9			N61S			E175K	E178K									
Line C plaque 10			N61S			E175K	E178K									
Line C plaque 11			N61S				E178K	G188R								
Line C plaque 12			N61S				E178K	G188R					T42I			
Line C plaque 14		T26I	N61S				E178K	G188R								
Line C plaque 15			N61S			E175K	E178K									

Corresponding lineage and plaque number are listed on the left. Gene within which each mutation was identified are listed at the top of the table. Mutation under the 'SUMO' label were identified within the SUMO tag appended to the N-terminus of *pylB*. Sub-populations with representative clusters of mutations were recloned into expression vectors for further analyses.

Supplementary Table 3. Mutations found within the *pylBCD* cassette in the evolved variants (sub-populations 1–6) and combinatorial variants (3f2, 3f2v2, JM10.1, 3f2v2+B, and 3f2+C).

Mutation		**S6L**	N61S	E84K	E122K	E175K	E178K	G188R	E251D	G332E	E315K	E316D	A252V	N61S	E84K
Old codon		TCG	AAC	GAG	GAG	GAG	GAG	GGG	GAA	GGA	GAA	GAA	GCT	AAC	GAG
New codon		TTG	AGC	AAG	AAG	AAG	AAG	CGG	GAC	GAA	AAA	GAT	GTT	AGC	AAG
Transition (TS) or Transversion (TV)		TS	TS	TS	TS	TS	TS	TV	TV	TS	TS	TV	TS	TS	TS
**Mutant Type**	**Name**	**SUMO**	**PylB**								**PylC**		**PylD**	**pBAD**	**pCDF**
Control	Empty	n/a	n/a	n/a	n/a	n/a	n/a	n/a	n/a	n/a	n/a	n/a	n/a	JH572	JH334
Wild-type	Untagged	n/a												JH573	JH20
PANCE parent	Tagged													JH462	JH126
Evolved (A2)	36A_subpop1		N61S		E122K		E178K	G188R						JH463	JH219
Evolved (A11)	36A_subpop2		N61S	E84K	E122K							E316D		JH574	JH220
Evolved (B2)	34B_subpop3	S6L	N61S				E178K	G188R	E251D				A252V	JH575	JH221
Evolved (B4)	34B_subpop4		N61S			E175K	E178K		E251D				A252V	JH576	JH222
Evolved (B15)	34B_subpop5		N61S				E178K		E251D				A252V	N/A	JH223
Evolved (C10)	40C_subpop6		N61S			E175K	E178K							N/A	JH224
Combinatorial	3f2		N61S	E84K	E122K		E178K	G188R	E251D				A252V	JH577	JH338
Combinatorial	3f2_v2		N61S	E84K	E122K		E178K	G188R	E251D			E316D	A252V	JH578	JH341
Combinatorial	JM10.1	S6L	N61S	E84K	E122K	E175K	E178K	G188R	E251D				A252V	JH579	JH405
Combinatorial	3f2v2+B		N61S	E84K	E122K		E178K	G188R	E251D	G332E		E316D	A252V	JH580	JH351
Combinatorial	3f2+C		N61S	E84K	E122K		E178K	G188R	E251D		E315K		A252V	JH581	JH359

Nucleotide sequences corresponding to each mutation are shown at the top of the table. On the far right, plasmid names for both pBAD and pCDF expression vectors containing the corresponding *pylBCD* variant are shown. Genotypes of evolved variants emerged in different plaques isolated following ALT-PANCE; combinatorial variants were cloned by rationally combining mutations identified in different plaques (see Text). A separate numbering scheme was used for the single mutation observed within the SUMO tag region of PylB (mutation S6L). For subsequent mutations in PylB, the first residue following the SUMO tag was designated residue ‘1’. As the start codon is included within the SUMO tag region, subsequent PylB residues are labeled with one number lower compared to the corresponding residue in WT PylB (eg., residue N61 here corresponds to residue N62 within the WT protein sequence).

Supplementary Table 4. Fragmentation table for LC-MS/MS spectra of sfGFP.2TAG with Pyl insertion confirmed at N39O.

B	B Ions	B+2H	B-NH3	B-H2O	AA	Y Ions	Y+2H	Y-NH3	Y-H2O	Y
1					S					15
2	187.11				V					14
3	343.21				R					13
4	400.23				G					12
5	529.27	265.14	512.25	511.26	E					11
6	586.29				G					10
7	715.34			697.33	E					9
8	772.36				G	898.5	449.75		880.49	8
9	887.39	444.2		869.37	D	841.48				7
10	958.42				A	726.45			708.44	6
11	1,059.47	530.24	1,042.44		T	655.41	328.21		637.4	5
12	1,296.62	648.81			O (N+123)	554.37				4
13		677.32			G	317.22	159.11			3
14		741.37			K	260.2		243.17		2
15					L					1

Masses of each B and Y ion are shown for each fragment. Masses of ions with an additional H^+, NH_3, and/or H_2O group are shown when detected. Amino acid residues corresponding to each fragment mass are indicated; O is used to indicate Pyl residues, with the amino acid and mass shift for the residue contained in the WT sfGFP sequence given in parenthesis.

Supplementary Table 5. Fragmentation table for LC-MS/MS spectra of sfGFP.2TAG with Pyl insertion confirmed at Y151O.

B	B Ions	B+2H	B-NH3	B-H2O	AA	Y Ions	Y+2H	Y-NH3	Y-H2O	Y
1					N					22
2	262.12		245.09		F					21
3			359.13		N					20
4					S		1,112.10			19
5	600.25		583.23		H					18
6	714.3			696.28	N	1,999.10				17
7	813.36				V	1,885.05				16
8	1,050.51			1,032.50	O (Y+74)					15
9	1,163.60	582.3	1,146.57		I					14
10		632.83		1,246.63	T	1,435.75	718.38		1,417.74	13
11	1,335.68			1,317.67	A	1,334.71				12
12	1,450.71			1,432.70	D	1,263.67				11
13	1,578.80	789.9			K	1,148.64		1,131.62		10
14			1,689.83		Q	1,020.55		1,003.52	1,002.54	9
15					K	892.49			874.48	8
16					N	764.39		747.37		7
17		1,004.01			G	649.37				6
18		1,060.55			I	592.35				5
19		1,124.60			K	479.26				4
20					A	351.17				3
21					N	280.13		263.1		2
22					F					1

Masses of each B and Y ion are shown for each fragment. Masses of ions with an additional H^+, NH_3, and/or H_2O group are shown when detected. Amino acid residues corresponding to each fragment mass are indicated; O is used to indicate Pyl residues, with the amino acid and mass shift for the residue contained in the WT sfGFP sequence given in parenthesis.

Supplementary Table 6. Codon usage analysis of PylB variants.

Evolved mutation site	S6L	N61S	E84K	E122K	E175K	E178K	G188R	E251D	Mutation site frequency total
SUMO-PylB codon	TCG	AAC	GAG	GAG	GAG	GAG	GGG	GAA	
SUMO-PylB codon frequency	12064	29304	24211	24211	24211	24211	14975	53822	207009
SUMO-PylB (opt) codon	AGC	AAC	GAA	GAA	GAA	GAA	GGC	GAA	
SUMO-PylB (opt) codon	21748	29304	53822	53822	53822	53822	40263	53822	360425
PylB.3f2 codon	TCG	AGC	AAG	AAG	GAG	AAG	AGG	GAC	
PylB.3f2 codon frequency	12064	21748	13937	13937	24211	13937	1491	25982	127307
PylB.3f2 (deopt) codon	TCG	TCA	AAG	AAG	GAG	AAG	AGG	GAC	
PylB.3f2 (deopt) codon frequency	12064	9620	13937	13937	24211	13937	1491	25982	115179
PylB.JM10.1 codon	TTG	AGC	AAG	AAG	AAG	AAG	AGG	GAC	
PylB.JM10.1 codon frequency	18488	21748	13937	13937	13937	13937	1491	25982	123457
PylB.JM10.1 (deopt) codon	CTA	TCA	AAG	AAG	AAG	AAG	AGG	GAC	
PylB.JM10.1 (deopt) codon	5260	9620	13937	13937	13937	13937	1491	25982	98101

For each PylB variant, codons used at each mutation site are shown. Below each codon, the number of instances (frequency) of the codon throughout the *E. coli* genome is also shown. Within the rightmost column, the sum of the frequencies for each codon within a given mutant are shown. As more frequently used codons are expected to be translated more efficiently, a higher mutation site frequency total is predicted to correlate with more efficient translation for a given mutant. For the synthetically prepared variant SUMO-PylB$_{opt}$, codons that were silently mutated to a more frequent codon are highlighted in blue. For the variants PyB.3f2$_{deopt}$ and PylB.JM10.1$_{deopt}$, codons that were silently mutated to a less frequent codon are highlighted in red. Codon frequency values were taken from Lajoie *et al* (2013)[4].

Supplementary Table 7. Melting temperature (Tm) values calculated from DSF experiments performed under varying salt conditions.

		Transition #1 Tm (°C)	Transition #2 Tm (°C)
**High Salt Samples (500 mM)**	SUMO-PylB (High salt)	54.6 ± 0.5	78 ± 0.4
	PylB.3f2 (High salt)	48.0 ± 3.4	70.4 ± 3.64
	PylB.JM10.1 (High salt)	48.8 ± 1.8	70 ± 2.4
**Low salt samples (10 mM)**	SUMO-PylB (Low salt)	50.6 ± 0.3	71.3 ± 0.6
	PylB.3f2 (Low salt)	48.4 ± 0.5	70.3 ± 1.0
	PylB.JM10.1 (Low salt)	48.1 ± 0.6	70.9 ± 1.4

Samples were tested without any additional additives, with the addition of SAM (10 mM), with the addition of L-lys (10 mM), and with the addition of both SAM and L-Lys. Tm values were determined using temperatures exhibiting maximal derivative fluorescence. For each sample shown above, both an early and a late transition were observed, with the Tm values shown for both. For the associated melting curves, see Supplementary Figure 13. Source data are provided as a Source Data file.

Supplementary Table 8. Melting temperature (Tm) values calculated from DSF experiments performed with different additives.

	Medium Salt Samples (350 mM)	Tm (°C)
No additives	SUMO-PylB (Medium salt)	80 ± 3.0
	PylB.3f2 (Medium salt)	81.7 ± 1.8
	PylB.JM10.1 (Medium salt)	81.7 ± 1.8
SAM added	SUMO-PylB (with SAM)	76.7 ± 0.6
	PylB.3f2 (with SAM)	78 ± 0.87
	PylB.JM10.1 (with SAM)	78 ± 0.87
L-Lys added	SUMO-PylB (with L-Lys)	77.3 ± 1
	PylB.3f2 (with L-Lys)	82.2 ± 1
	PylB.JM10.1 (with L-Lys)	81.1 ± 3.1
SAM and L-Lys added	SUMO-PylB (with SAM and L-Lys)	80.5 ± 1.3
	PylB.3f2 (with SAM and L-Lys)	76.5 ± 1.3
	PylB.JM10.1 (with SAM and L-Lys)	82.8 ± 4

For each sample, Tm values were determined using temperatures exhibiting maximal derivative fluorescence. Experiments were performed under medium salt conditions (350 mM). For the associated melting curves, see Supplementary Figure 14. Source data are provided as a Source Data file.

Supplementary Table 9. Description of plasmids deposited in Addgene.

Plasmid	Purpose	Insert	Mutation	Resistance	Origin
JH19	Accessory Plasmid for PACE of pylBCD variants	Ppsp gIII.1TAG, luxAB; Plpp pylS; PproK pylT	gIII(P29*)	Spectinomycin	ColE1
JH60	Microscopy inclusion body assay	pCDF.CD	PylCD partial operon (PylB deleted to assess impact on inclusion body formation)	Spectinomycin	pBR322
JH61	Accessory Plasmid for PACE of pylBCD variants	Ppsp gIII.1TAG, luxAB; Plpp [eG SD8] pylS; PproK pylT	gIII(P29*); [strong promoter eG, strong RBS SD8] pylS	Spectinomycin	ColE1
JH76	Accessory Plasmid for PACE of pylBCD variants	Ppsp gIII.2TAG, luxAB; Plpp pylS; PproK pylT	gIII(P29*, Y184*)	Spectinomycin	ColE1
JH77	Accessory Plasmid for PACE of pylBCD variants	Ppsp gIII.3TAG, luxAB; Plpp pylS; PproK pylT	gIII(P29*, P83*, Y184*)	Spectinomycin	ColE1
JH123	Microscopy inclusion body assay	pCDF.B.(SD4)CD	PylBCD operon (RBS inserted for PylC)	Spectinomycin	pBR322
JH126	Microscopy inclusion body assay	pCDF.SUMO-B.(SD4).CD	PylBCD operon (SUMO tag inserted for PylB; RBS inserted for PylC)	Spectinomycin	pBR322
JH190	Accessory Plasmid for PACE of pylBCD variants	Ppsp gIII.1TAG, luxAB; Plpp [eG SD8] 32A-Nter; PproK pylT	gIII(P29*); [strong promoter eG, strong RBS SD8] 32A-Nter pylS variant	Spectinomycin	ColE1
JH191	Accessory Plasmid for PACE of pylBCD variants	Ppsp gIII.2TAG, luxAB; Plpp [eG SD8] 32A-Nter; PproK pylT	gIII(P29*, Y184*); [strong promoter eG, strong RBS SD8] 32A-Nter pylS variant	Spectinomycin	ColE1
JH202	Accessory Plasmid for PACE of pylBCD variants	Ppsp gIII.1TAG, luxAB; Plpp 32A; PproK pylT	gIII(P29*); 32A pylS variant	Spectinomycin	ColE1
JH223	pylBCD variant in pCDF backbone	PT7/lac (pylBCD 34B_sub-pop5); LacI	PylB(N61S, E178K, E251D) PylD(A252V)	Spectinomycin	pBR322
JH224	pylBCD variant in pCDF backbone	PT7/lac (pylBCD 40C_sub-pop6); LacI	PylB(N61S, E175K, E178K)	Spectinomycin	pBR322
JH291	Reporter plasmid for pylBCD variant in pCDF backbone	Para sfGFP.2TAG; Plpp pylS; PproK pylT; AraC	sfGFP(N39*, Y151*)	Ampicillin	p15A
JH462	pylBCD variant in pBAD backbone	Para (Sumo-tagged pylBCD); AraC	SUMO tag fused to N-terminus of PylB	Ampicillin	pBR322
JH463	pylBCD variant in pBAD backbone	Para (pylBCD 36A_sub-pop1); AraC	PylB(N61S, E122K, E178K, G188R)	Ampicillin	pBR322
JH572	Negative control pBAD backbone	pBAD backbone	No insert	Ampicillin	pBR322
JH573	pylBCD variant in pBAD backbone	Para (Untagged pylBCD); AraC	Codon-optimized Methanosarcina acetivorans pylBCD	Ampicillin	pBR322
JH574	pylBCD variant in pBAD backbone	Para (pylBCD 36A_sub-pop2); AraC	PylB(N61S, E84K, E122K) PylC(E316D)	Ampicillin	pBR322
JH575	pylBCD variant in pBAD backbone	Para (pylBCD 34B_sub-pop3); AraC	Sumo(S6L) PylB(N61S, E178K, G188R, E251D) PylD(A252V)	Ampicillin	pBR322
JH576	pylBCD variant in pBAD backbone	Para (pylBCD 34B_sub-pop4); AraC	PylB(N61S, E175K, E178K, E251D) PylD(A252V)	Ampicillin	pBR322
JH577	pylBCD variant in pBAD backbone	Para (pylBCD 3f2); AraC	PylB(N61S, E84K, E122K, E178K, G188R, E251D) PylD(A252V)	Ampicillin	pBR322
JH578	pylBCD variant in pBAD backbone	Para (pylBCD 3f2_v2); AraC	PylB(N61S, E84K, E122K, E178K, G188R, E251D) PylC(E316D) PylD(A252V)	Ampicillin	pBR322
JH579	pylBCD variant in pBAD backbone	Para (pylBCD JM10.1); AraC	Sumo(S6L) PylB(N61S, E84K, E122K, E175K, E178K, G188R, E251D) PylD(A252V)	Ampicillin	pBR322
JH580	pylBCD variant in pBAD backbone	Para (pylBCD 3f2v2+B); AraC	PylB(N61S, E84K, E122K, E178K, G188R, E251D, G332E) PylC(E316D) PylD(A252V)	Ampicillin	pBR322
JH581	pylBCD variant in pBAD backbone	Para (pylBCD 3f2+C); AraC	PylB(N61S, E84K, E122K, E178K, G188R, E251D) PylC(E315K) PylD(A252V)	Ampicillin	pBR322
JH528	Reporter plasmid for pylBCD variant in pBAD backbone	Para sfGFP.1TAG; Plpp pylS; PproK pylT; AraC	sfGFP(N39*)	Tetracycline	p15A
JH530	Reporter plasmid for pylBCD variant in pBAD backbone	Para sfGFP.3TAG; Plpp pylS; PproK pylT; AraC; Tet	sfGFP(N39*, N135*, Y151*)	Tetracycline	p15A
JH643	Reporter plasmid for pylBCD variant in pBAD backbone	Para sfGFP.3TAG; Plpp pylS; PproK pylT; AraC; Cam	sfGFP(N39*, N135*, Y151*)	Chloramphenicol	p15A
JH645	Reporter plasmid for pylBCD variant in pBAD backbone	Pem7 luxCDA(B.1TAG)E; Plpp pylS; PproK pylT	Inserted amber codon immediately after initiator methionine of luxB	Chloramphenicol	p15A
JH750	Microscopy inclusion body assay	pCDF.3f2, deleted sumo	Evolved PylBCD operon; SUMO tag deleted from PylB [PylB(N61S, E84K, E122K, E178K, G188R, E251D) PylD(A252V)]	Spectinomycin	pBR322
JH751	Microscopy inclusion body assay	pCDF.JM10.1, deleted sumo	Evolved PylBCD operon; SUMO tag deleted from PylB [PylB(N61S, E84K, E122K, E175K, E178K, G188R, E251D) PylD(A252V)]	Spectinomycin	pBR322
JH767	PylB overexpression and purification	PT7/Lac-[His6-TEV-SUMO-PylB.WT]	pET28a_LacI_PT7/Lac-[His6-TEV-SUMO-PylB]	Kanamycin	pBR322
JH768v2	PylB overexpression and purification	PT7/Lac-[His6-TEV-SUMO-PylB.3f2]	pET28a_LacI_PT7/Lac-[His6-TEV-SUMO-PylB(N61S, E84K, E122K, E178K, G188R, E251D)]	Kanamycin	pBR322
JH769v2	PylB overexpression and purification	PT7/Lac-[His6-TEV-SUMO-PylB.JM10.1]	pET28a_LacI_PT7/Lac-[His6-TEV-SUMO-PylB(N61S, E84K, E122K, E175K, E178K, G188R, E251D)]	Kanamycin	pBR322
JH857	PylB overexpression and purification	PT7/Lac-[His6-TEV-SUMO-PylB.WT_{opt}]	pET28a_LacI_PT7/Lac-[His6-TEV-SUMO-$PylB_{opt}$]	Kanamycin	pBR322
JH858	PylB overexpression and purification	PT7/Lac-[His6-TEV-SUMO-PylB.$3f2_{deopt}$]	pET28a_LacI_PT7/Lac-[His6-TEV-SUMO-$PylB_{deopt}$(N61S, E84K, E122K, E178K, G188R, E251D)]	Kanamycin	pBR322
JH859	PylB overexpression and purification	PT7/Lac-[His6-TEV-SUMO-PylB.$JM10.1_{deopt}$]	pET28a_LacI_PT7/Lac-[His6-TEV-SUMO-$PylB_{deopt}$(N61S, E84K, E122K, E175K, E178K, G188R, E251D)]	Kanamycin	pBR322

Supplementary references

1. Gaston, M.A., Zhang, L., Green-Church, K.B. & Krzycki, J.A. (2011) The complete biosynthesis of the genetically encoded amino acid pyrrolysine from lysine. *Nature* **471**, 647-650.
2. Ho, J.M. *et al*. (2016) Efficient reassignment of a frequent serine codon in wild-type *Escherichia coli. ACS Synth Biol* **5**, 163-171.
3. Suzuki, T. *et al*. (2017) Crystal structures reveal an elusive functional domain of pyrrolysyl-tRNA synthetase. *Nat Chem Biol* **13**, 1261-1266.
4. Lajoie, M.J. *et al*. (2013) Probing the limits of genetic recoding in essential genes. *Science* **342**, 361-363.
