# Supplementary material for: Improved pyrrolysine biosynthesis through phage assisted non-continuous directed evolution of the complete pathway
Source: Nat Commun. 2021 Jun 24;12:3914. doi: 10.1038/s41467-021-24183-9 (PMC8225853; doi:10.1038/s41467-021-24183-9)
Supplement: Supplementary file 3 — Description of Additional Supplementary Files [file 41467_2021_24183_MOESM3_ESM.pdf]

Description of additional supplementary files

Title: Supplementary Data 1
Description: DNA Sequences of wild-type and codon-optimized Methanosarcina acetivorans pylBCD variants used in this study
