# Supplementary material for: Improved pyrrolysine biosynthesis through phage assisted non-continuous directed evolution of the complete pathway
Source: Nat Commun. 2021 Jun 24;12:3914. doi: 10.1038/s41467-021-24183-9 (PMC8225853; doi:10.1038/s41467-021-24183-9)
Supplement: Supplementary file 4 — Reporting Summary [file 41467_2021_24183_MOESM4_ESM.pdf]

nature research

Corresponding author(s): Matthew R. Bennett
Last updated by author(s): Jun 1, 2021

Reporting Summary

Nature Research wishes to improve the reproducibility of the work that we publish. This form provides structure for consistency and transparency in reporting. For further information on Nature Research policies, see our Editorial Policies and the Editorial Policy Checklist.

Statistics

For all statistical analyses, confirm that the following items are present in the figure legend, table legend, main text, or Methods section.

n/a	Confirmed	
☐	☒	The exact sample size (n) for each experimental group/condition, given as a discrete number and unit of measurement
☐	☒	A statement on whether measurements were taken from distinct samples or whether the same sample was measured repeatedly
☐	☒	The statistical test(s) used AND whether they are one- or two-sided<br>*Only common tests should be described solely by name; describe more complex techniques in the Methods section.*
☒	☐	A description of all covariates tested
☒	☐	A description of any assumptions or corrections, such as tests of normality and adjustment for multiple comparisons
☐	☒	A full description of the statistical parameters including central tendency (e.g. means) or other basic estimates (e.g. regression coefficient) AND variation (e.g. standard deviation) or associated estimates of uncertainty (e.g. confidence intervals)
☐	☒	For null hypothesis testing, the test statistic (e.g. F, t, r) with confidence intervals, effect sizes, degrees of freedom and P value noted<br>*Give P values as exact values whenever suitable.*
☒	☐	For Bayesian analysis, information on the choice of priors and Markov chain Monte Carlo settings
☒	☐	For hierarchical and complex designs, identification of the appropriate level for tests and full reporting of outcomes
☒	☐	Estimates of effect sizes (e.g. Cohen's d, Pearson's r), indicating how they were calculated

Our web collection on statistics for biologists contains articles on many of the points above.

Software and code

Policy information about availability of computer code

Data collection	Bio-Rad CFX Manager v3.1, Nikon NIS-Elements v4.51
Data analysis	Image J v1.47, LI-COR Image Studio v5.2, Microsoft Excel v16.47.1 , Prism v9.1.0

For manuscripts utilizing custom algorithms or software that are central to the research but not yet described in published literature, software must be made available to editors and reviewers. We strongly encourage code deposition in a community repository (e.g. GitHub). See the Nature Research guidelines for submitting code & software for further information.

Data

Policy information about availability of data

All manuscripts must include a data availability statement. This statement should provide the following information, where applicable:

- Accession codes, unique identifiers, or web links for publicly available datasets
- A list of figures that have associated raw data
- A description of any restrictions on data availability

Raw data from our DSF (Thermofluor), proteolytic degradation, and Western blot experiments are available in the Source Data file. All other raw data is available from the corresponding author upon request. Addgene repository URL is specified, as was requested in the Editorial Request document.

Field-specific reporting

Please select the one below that is the best fit for your research. If you are not sure, read the appropriate sections before making your selection.

☒ Life sciences ☐ Behavioural & social sciences ☐ Ecological, evolutionary & environmental sciences

For a reference copy of the document with all sections, see nature.com/documents/nr-reporting-summary-flat.pdf

Life sciences study design

All studies must disclose on these points even when the disclosure is negative.

Sample size	All continuous evolution and biochemical tests were performed in at least three biological replicates. Sample size is defined as the specific number of physically distinct colonies (separate samples stored in separate containers), and three was the number chosen because it is the standard number of biological replicates that is typically used in biochemical assays.
Data exclusions	No data were excluded.
Replication	All biochemical tests were repeated at least 3 times, with all attempts successful.
Randomization	Not applicable, because samples were labeled with arbitrary numbers in all assays.
Blinding	Not applicable, because samples were labeled with arbitrary numbers in all assays.

Reporting for specific materials, systems and methods

We require information from authors about some types of materials, experimental systems and methods used in many studies. Here, indicate whether each material, system or method listed is relevant to your study. If you are not sure if a list item applies to your research, read the appropriate section before selecting a response.

Materials & experimental systems

n/a	Involved in the study
☐	☒ Antibodies
☒	☐ Eukaryotic cell lines
☒	☐ Palaeontology and archaeology
☒	☐ Animals and other organisms
☒	☐ Human research participants
☒	☐ Clinical data
☒	☐ Dual use research of concern

Methods

n/a	Involved in the study
☒	☐ ChIP-seq
☒	☐ Flow cytometry
☒	☐ MRI-based neuroimaging

Antibodies

Antibodies used	Mouse anti-GAPDH antibody (Invitrogen MA5-15738), clone GA1R, RRID AB_10977387, lot VF300724; Mouse anti-His antibody (ThermoFisher MA121315), clone HIS.H8, RRID AB_557403, lot OE186264; Goat anti-mouse antibody (LI-COR 926-68070), polyclonal, RRID AB_10956588, lot C90910-21.
Validation	MA5-15738 has been successfully used in Western blot, ICC, IF, IHC (P), FACS and ELISA; has been used to detect GAPDH from BL-21 bacteria, Sf9 insect, yeast, human, mouse, rat, rabbit, hamster, and chicken samples. MA121315 was verified by relative expression to ensure that the antibody binds to the antigen stated. LI-COR 926-68070 was tested by Dot Blot, solid-phase adsorbed for minimal cross-reactivity with human, rabbit, goat, rat, and horse serum proteins, and has been specifically tested and qualified for Western blot and In-Cell Western applications.
