# Supplementary material for: Improved pyrrolysine biosynthesis through phage assisted non-continuous directed evolution of the complete pathway
Source: Nat Commun. 2021 Jun 24;12:3914. doi: 10.1038/s41467-021-24183-9 (PMC8225853; doi:10.1038/s41467-021-24183-9)
Supplement: Supplementary file 5 — Source Data [file 41467_2021_24183_MOESM5_ESM.zip › Source Data_REV 2/Figure 4A_4B_Source.docx]

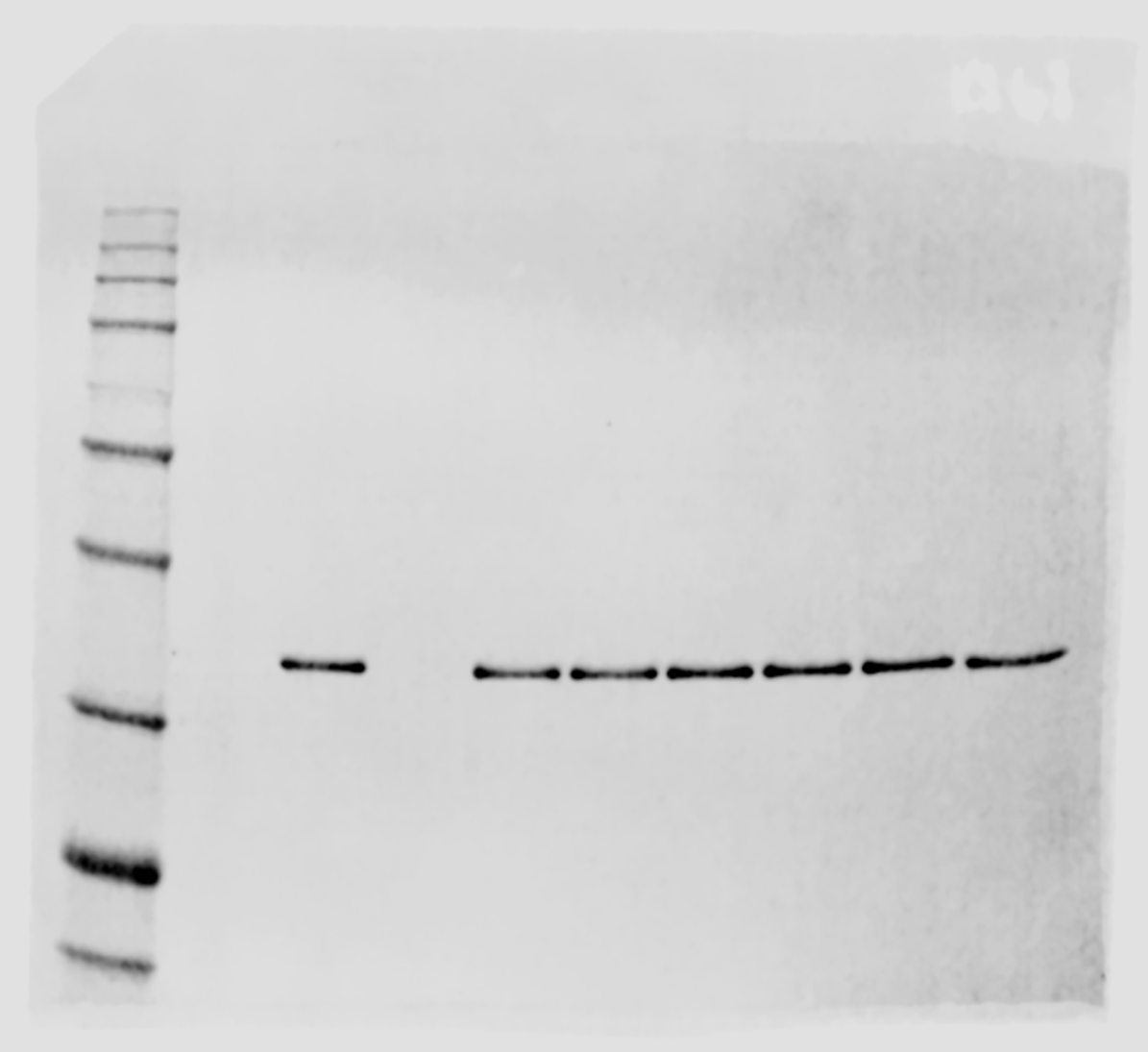


**PylB sample Western blot replicate #1, treated using GAPDH antibody only (housekeeping gene).** Samples loaded in each lane (from left to right) are (1) Protein ladder (2) empty lane (3) Cell prep using empty plasmid vector (4) empty lane (5) SUMO-PylB (6) PylB.3f2 (7) PylB.JM10.1 (8) SUMO-PylB_opt_ (9) PylB.3f2_deopt_ (10) PylB.JM10.1_deopt_.

**
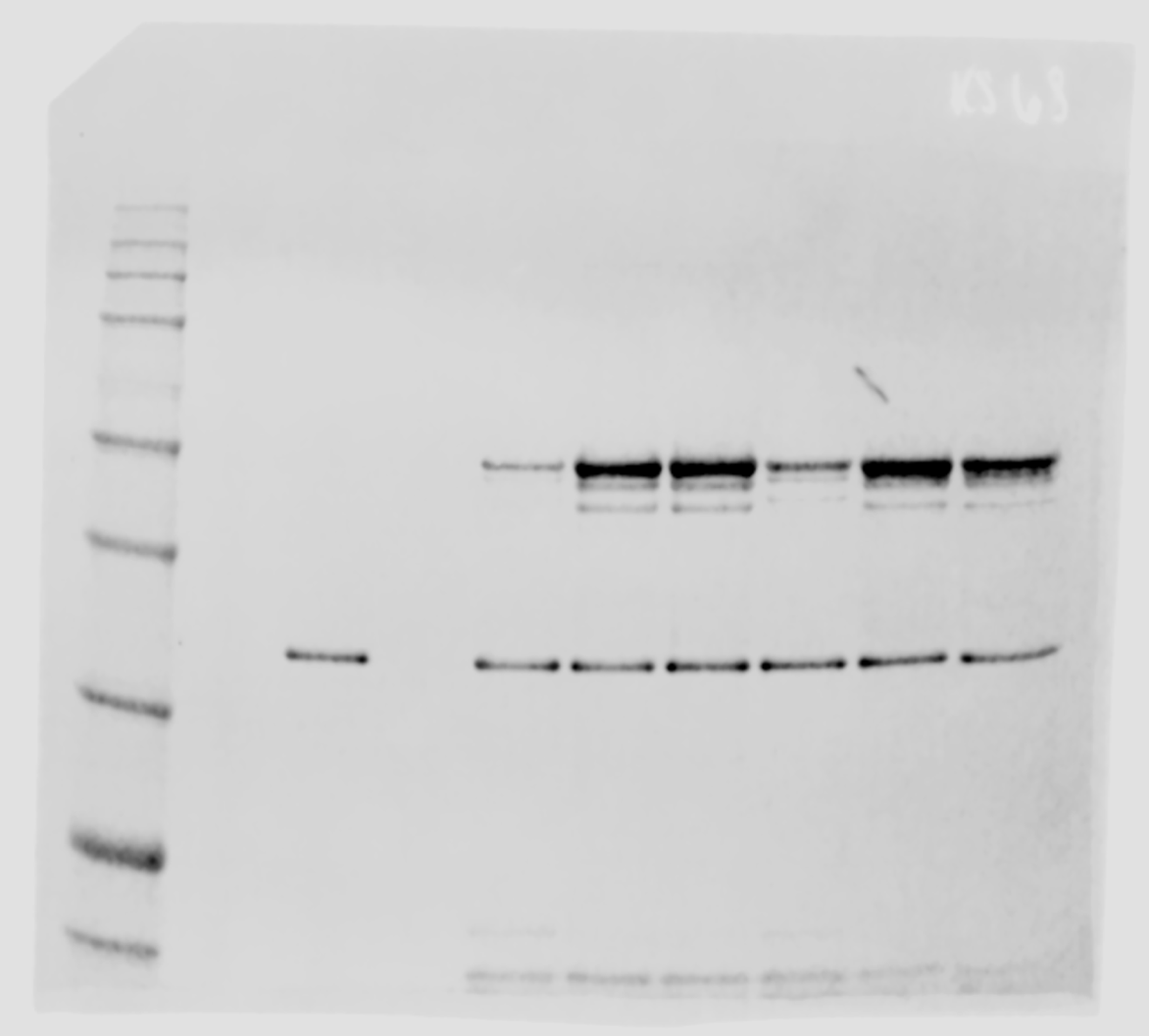
**

**PylB sample Western blot replicate #1, treated using His tag antibody (upper bands) and GAPDH antibody (lower bands).** Samples loaded in each lane (from left to right) are (1) Protein ladder (2) empty lane (3) Cell prep using empty plasmid vector (4) empty lane (5) SUMO-PylB (6) PylB.3f2 (7) PylB.JM10.1 (8) SUMO-PylB_opt_ (9) PylB.3f2_deopt_ (10) PylB.JM10.1_deopt_.

**
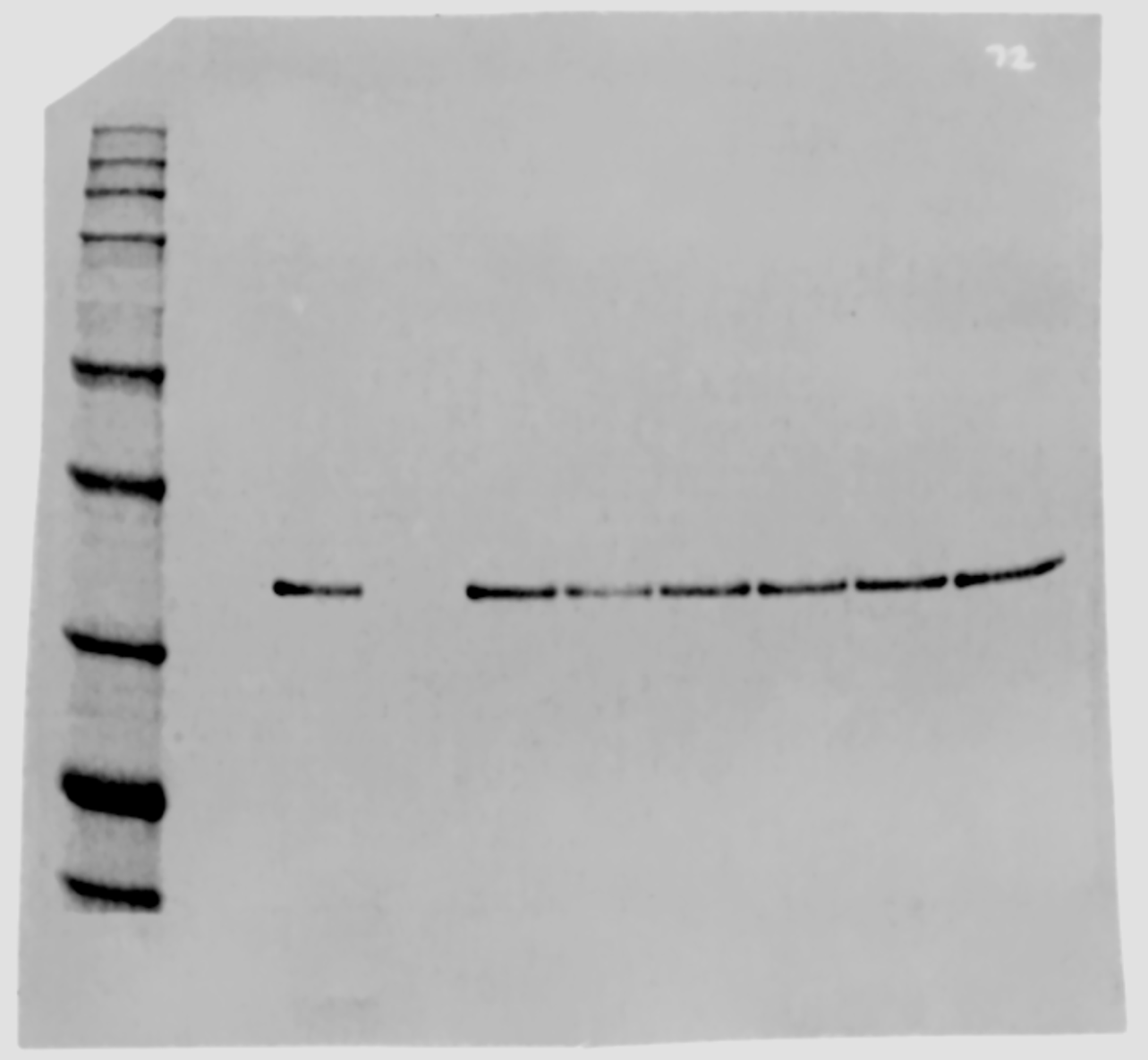
**

**PylB sample Western blot replicate #2, treated using GAPDH antibody only (housekeeping gene).** Samples loaded in each lane (from left to right) are (1) Protein ladder (2) empty lane (3) Cell prep using empty plasmid vector (4) empty lane (5) SUMO-PylB (6) PylB.3f2 (7) PylB.JM10.1 (8) SUMO-PylB_opt_ (9) PylB.3f2_deopt_ (10) PylB.JM10.1_deopt_.


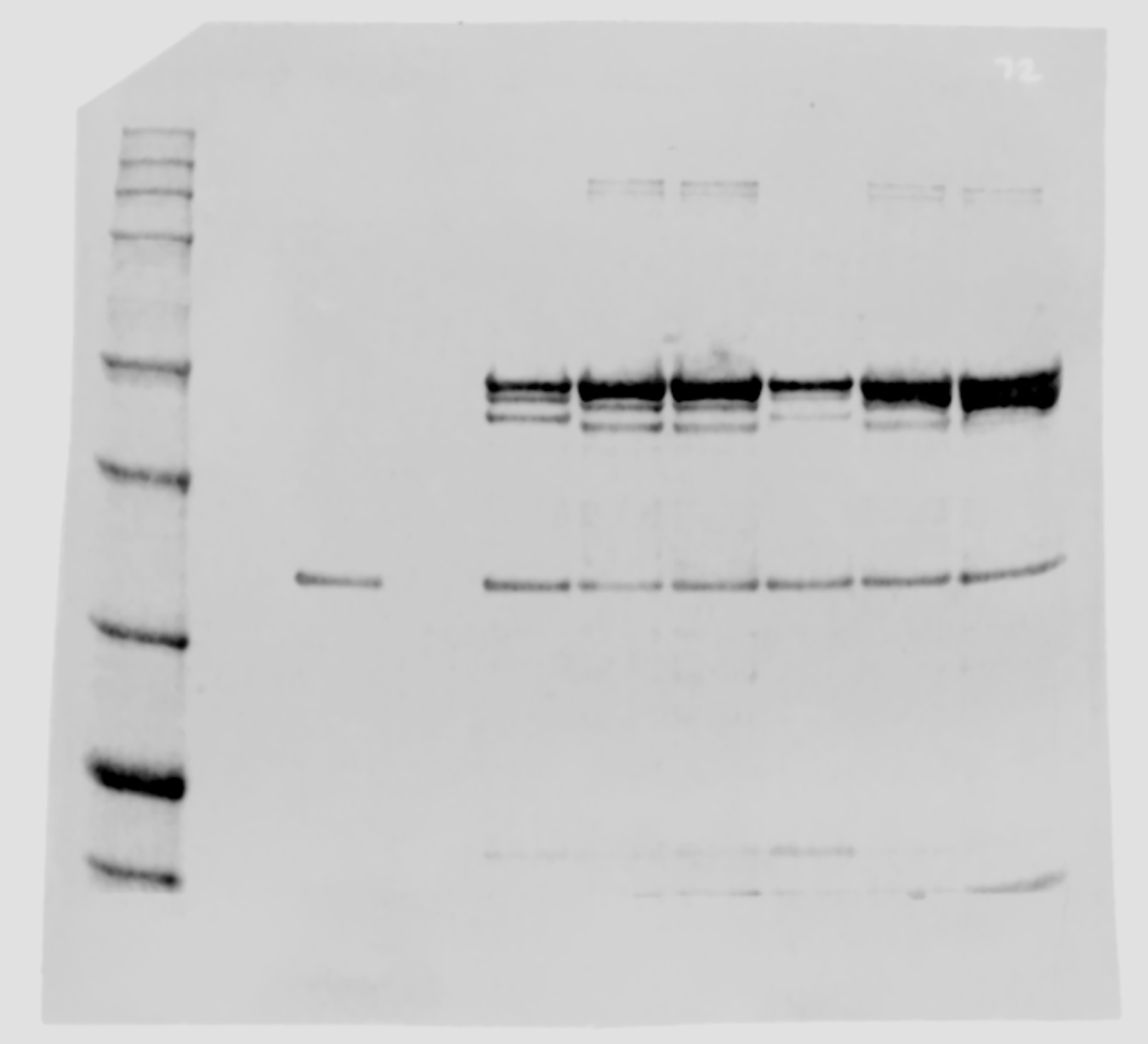


**PylB sample Western blot replicate #2, treated using His tag antibody (upper bands) and GAPDH antibody (lower bands).** Samples loaded in each lane (from left to right) are (1) Protein ladder (2) empty lane (3) Cell prep using empty plasmid vector (4) empty lane (5) SUMO-PylB (6) PylB.3f2 (7) PylB.JM10.1 (8) SUMO-PylB_opt_ (9) PylB.3f2_deopt_ (10) PylB.JM10.1_deopt_.

**
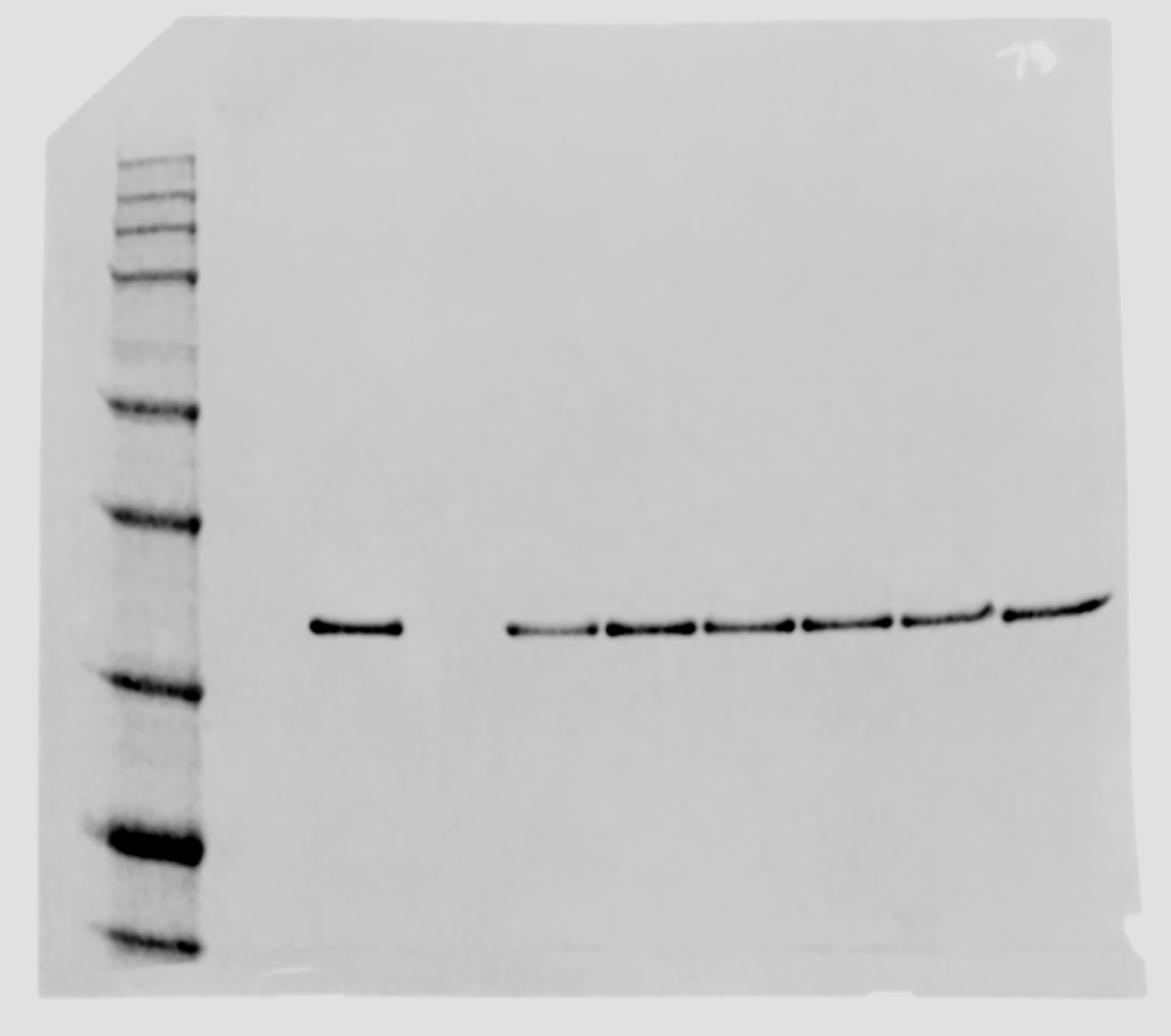
**

**PylB sample Western blot replicate #3, treated using GAPDH antibody only (housekeeping gene).** Samples loaded in each lane (from left to right) are (1) Protein ladder (2) empty lane (3) Cell prep using empty plasmid vector (4) empty lane (5) SUMO-PylB (6) PylB.3f2 (7) PylB.JM10.1 (8) SUMO-PylB_opt_ (9) PylB.3f2_deopt_ (10) PylB.JM10.1_deopt_.


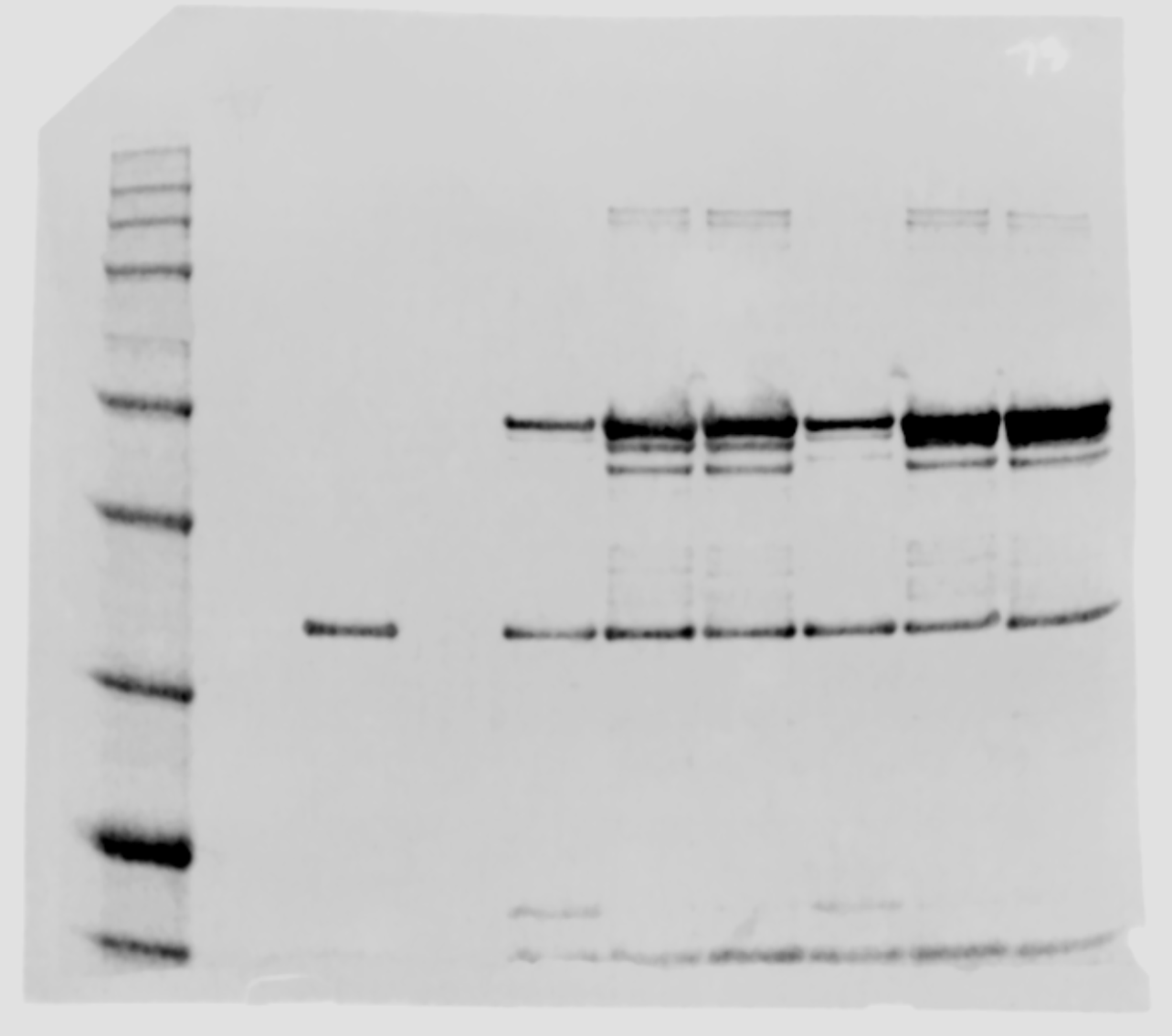


**PylB sample Western blot replicate #3, treated using His tag antibody (upper bands) and GAPDH antibody (lower bands).** Samples loaded in each lane (from left to right) are (1) Protein ladder (2) empty lane (3) Cell prep using empty plasmid vector (4) empty lane (5) SUMO-PylB (6) PylB.3f2 (7) PylB.JM10.1 (8) SUMO-PylB_opt_ (9) PylB.3f2_deopt_ (10) PylB.JM10.1_deopt_.


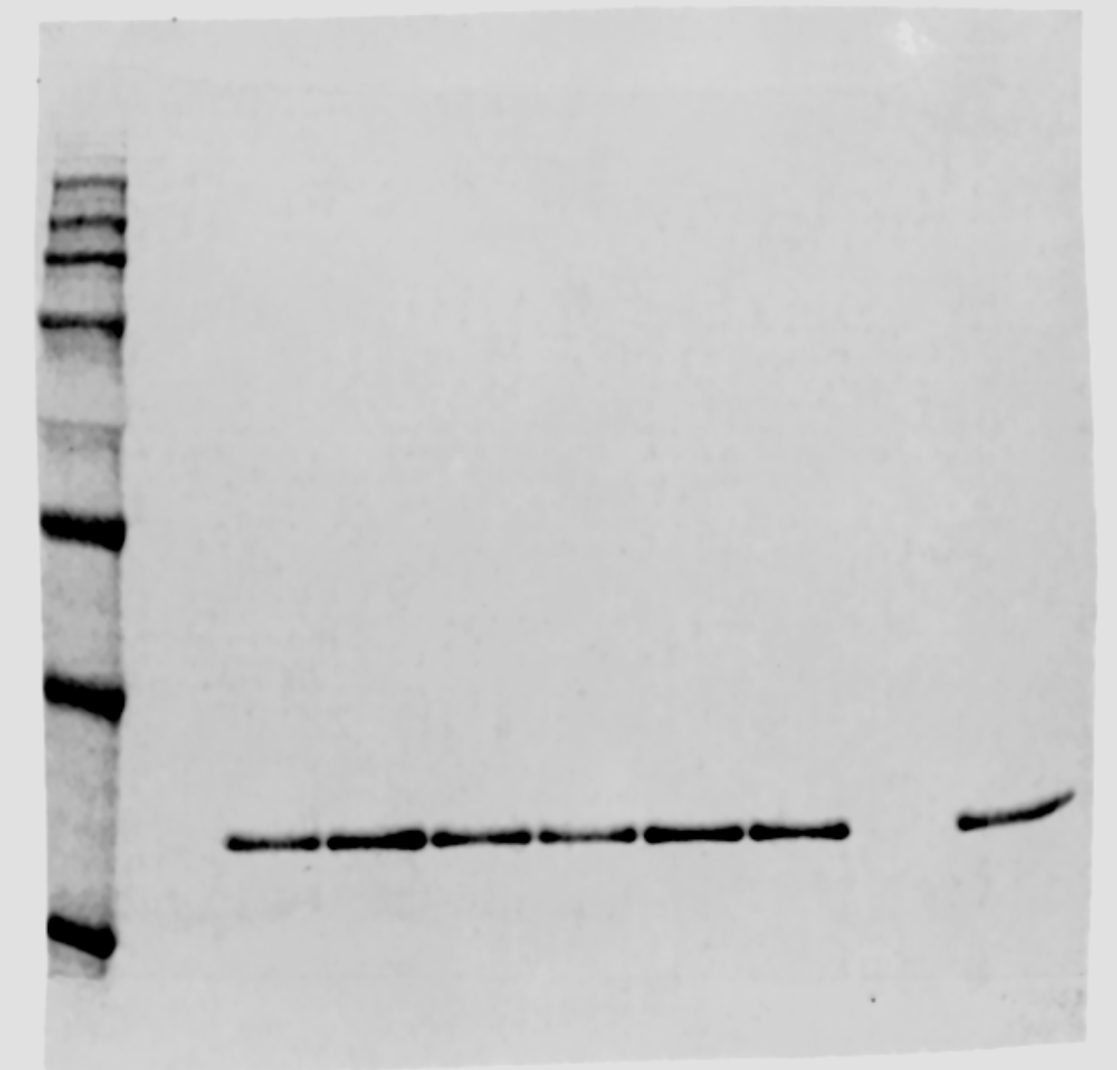


**PylB sample Western blot replicate #4, treated using GAPDH antibody only (housekeeping gene).** Samples loaded in each lane (from left to right) are (1) Protein ladder (2) empty lane (3) Cell prep using empty plasmid vector (4) empty lane (5) SUMO-PylB (6) PylB.3f2 (7) PylB.JM10.1 (8) SUMO-PylB_opt_ (9) PylB.3f2_deopt_ (10) PylB.JM10.1_deopt_.

**
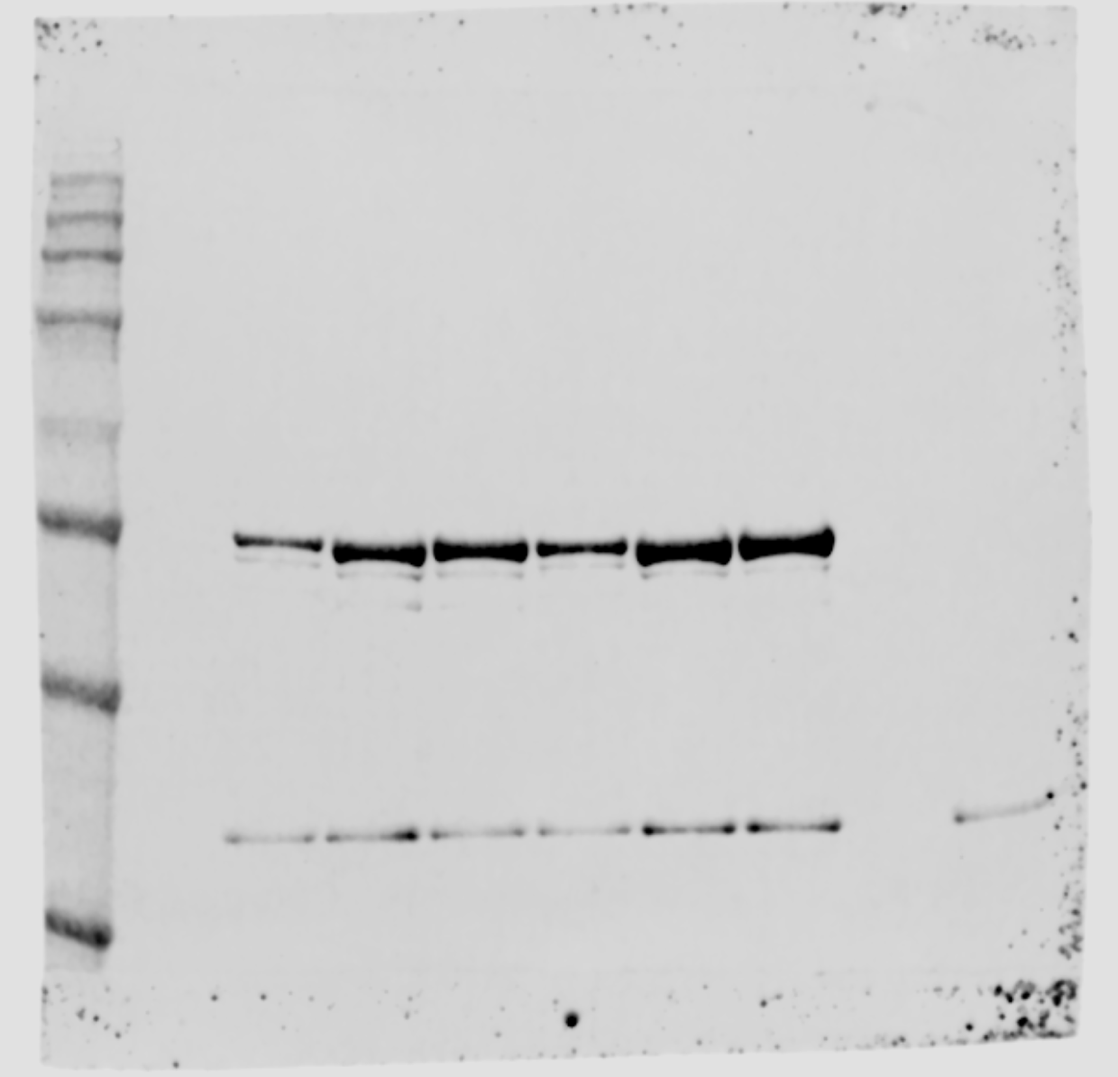
**

**PylB sample Western blot replicate #4, treated using His tag antibody (upper bands) and GAPDH antibody (lower bands).** Samples loaded in each lane (from left to right) are (1) Protein ladder (2) empty lane (3) Cell prep using empty plasmid vector (4) empty lane (5) SUMO-PylB (6) PylB.3f2 (7) PylB.JM10.1 (8) SUMO-PylB_opt_ (9) PylB.3f2_deopt_ (10) PylB.JM10.1_deopt_.


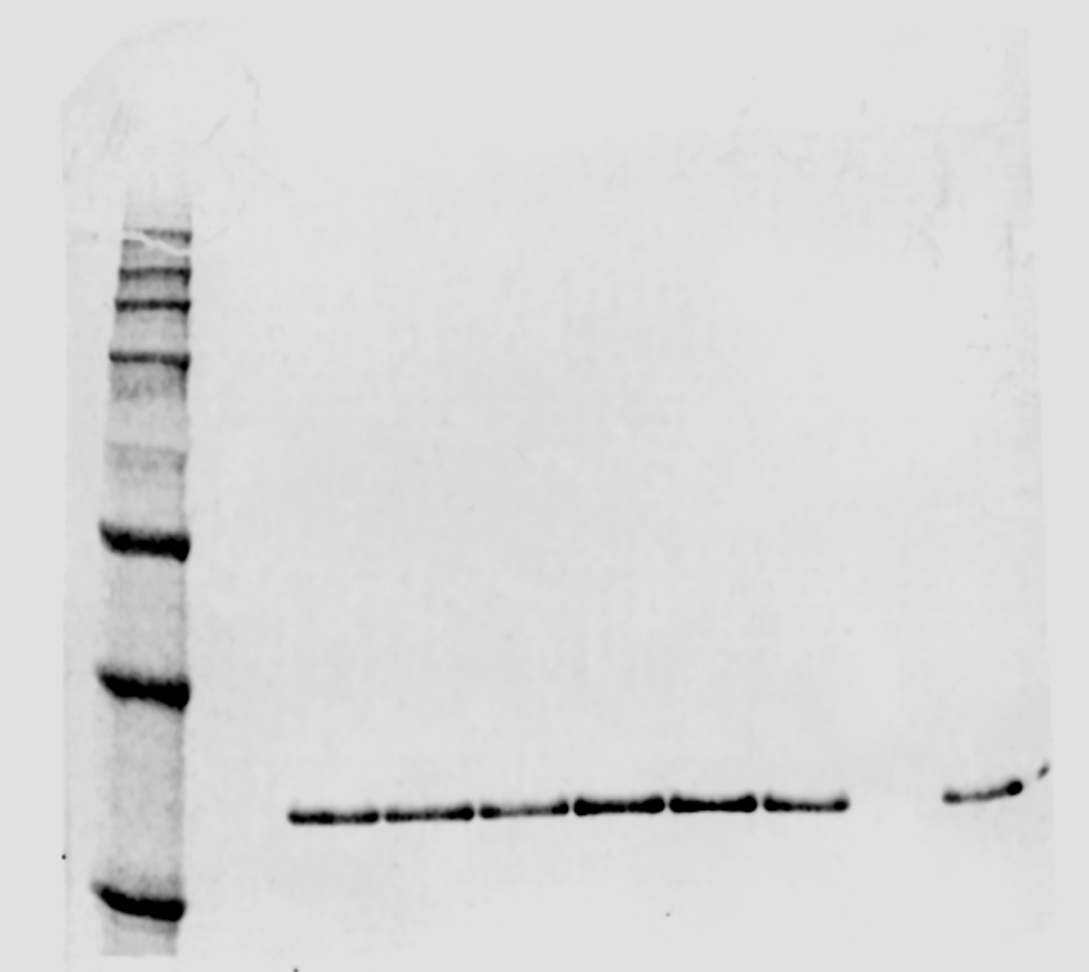


**PylB sample Western blot replicate #5, treated using GAPDH antibody only (housekeeping gene).** Samples loaded in each lane (from left to right) are (1) Protein ladder (2) empty lane (3) Cell prep using empty plasmid vector (4) empty lane (5) SUMO-PylB (6) PylB.3f2 (7) PylB.JM10.1 (8) SUMO-PylB_opt_ (9) PylB.3f2_deopt_ (10) PylB.JM10.1_deopt_.


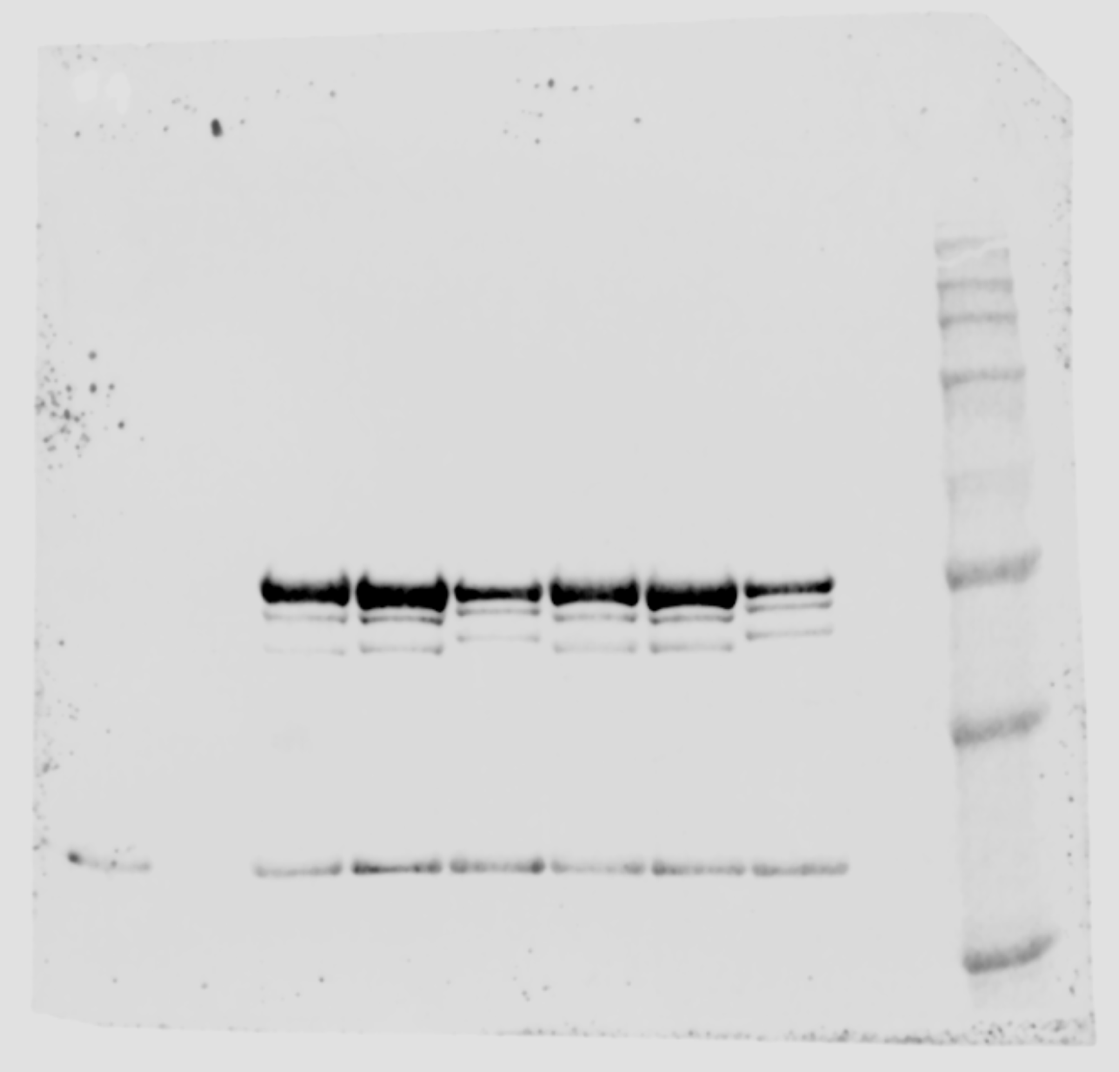


**PylB sample Western blot replicate #5, treated using His tag antibody (upper bands) and GAPDH antibody (lower bands).** Samples loaded in each lane (from left to right) are (1) Protein ladder (2) empty lane (3) Cell prep using empty plasmid vector (4) empty lane (5) SUMO-PylB (6) PylB.3f2 (7) PylB.JM10.1 (8) SUMO-PylB_opt_ (9) PylB.3f2_deopt_ (10) PylB.JM10.1_deopt_.


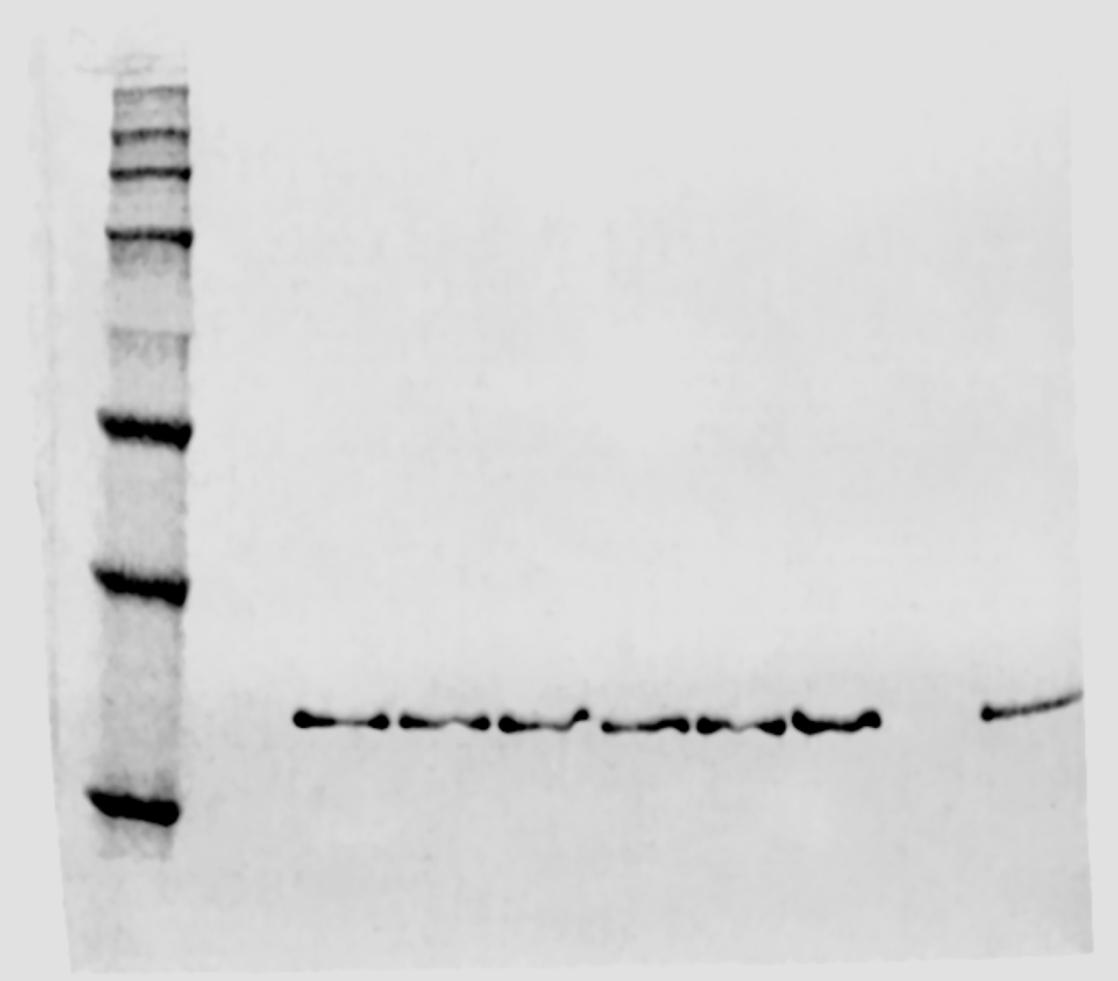


**PylB sample Western blot replicate #6, treated using GAPDH antibody only (housekeeping gene).** Samples loaded in each lane (from left to right) are (1) Protein ladder (2) empty lane (3) Cell prep using empty plasmid vector (4) empty lane (5) SUMO-PylB (6) PylB.3f2 (7) PylB.JM10.1 (8) SUMO-PylB_opt_ (9) PylB.3f2_deopt_ (10) PylB.JM10.1_deopt_.

**
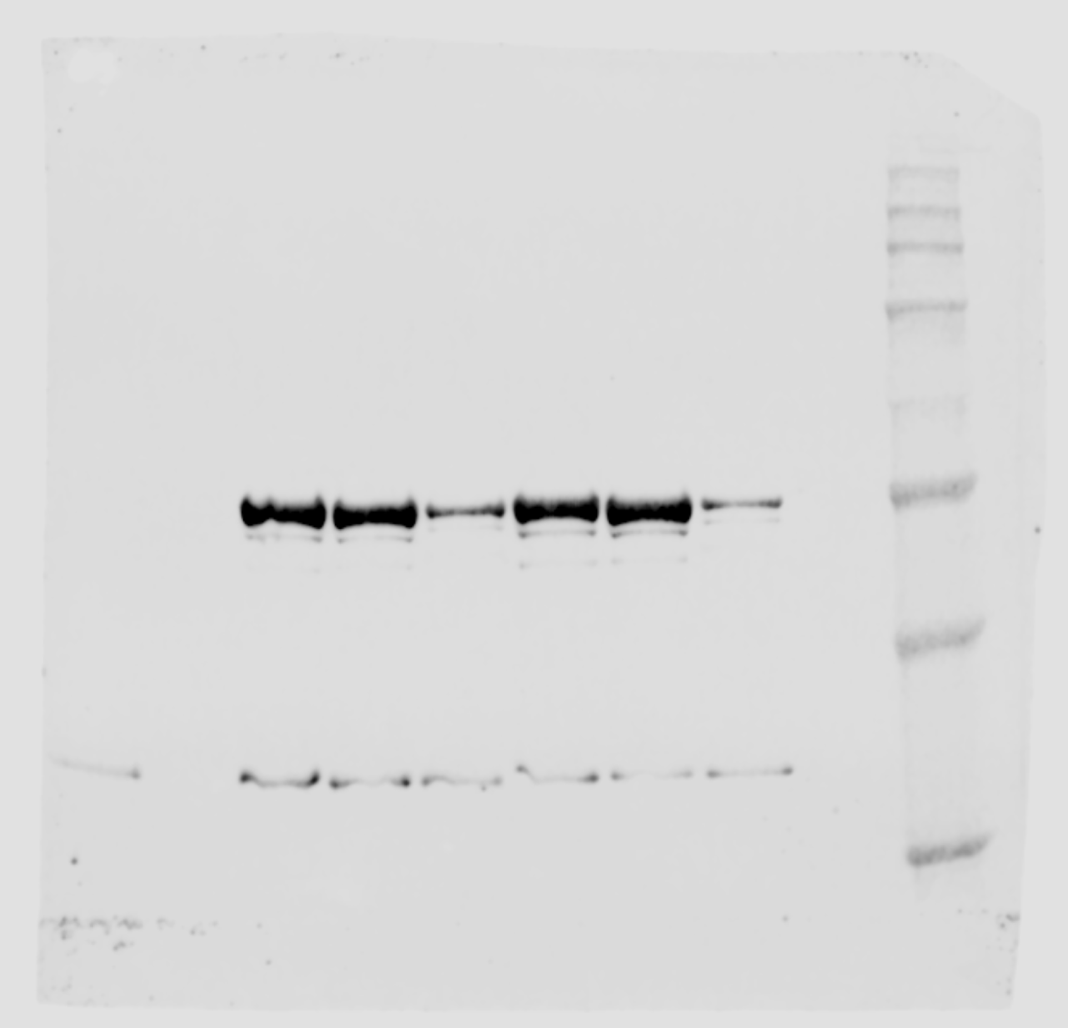
**

**PylB sample Western blot replicate #5, treated using His tag antibody (upper bands) and GAPDH antibody (lower bands).** Samples loaded in each lane (from left to right) are (1) Protein ladder (2) empty lane (3) Cell prep using empty plasmid vector (4) empty lane (5) SUMO-PylB (6) PylB.3f2 (7) PylB.JM10.1 (8) SUMO-PylB_opt_ (9) PylB.3f2_deopt_ (10) PylB.JM10.1_deopt_.


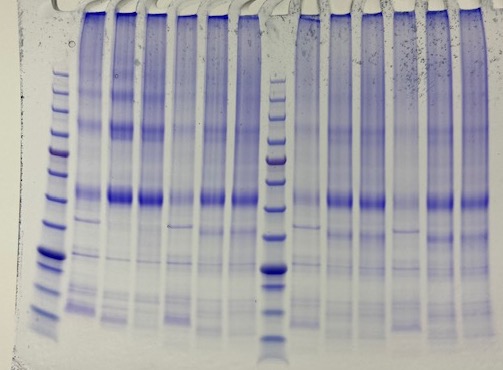


**Chymotrypsin proteolysis gel image #1**. Samples loaded in each lane (from left to right) are: (1) Bluestain 2 Protein Ladder, 4 µL loaded (2) SUMO-PylB, undigested, 8 µL loaded, replicate #1 (3) PylB.3f2, undigested, 8 µL loaded, replicate #1 (4) PylB.JM10.1, undigested, 8 µL loaded, replicate #1 (5) SUMO-PylB, 30 second digest, 8 µL loaded, replicate #1 (6) PylB.3f2, 30 second digest, 8 µL loaded, replicate #1 (7) PylB.JM10.1, 30 second digest, 8 µL loaded, replicate #1 (8) Bluestain 2 Protein Ladder, 4 µL loaded (9) SUMO-PylB, 1 min digest, 8 µL loaded, replicate #1 (10) PylB.3f2, 1 min digest, 8 µL loaded, replicate #1 (11) PylB.JM10.1, 1 min digest, 8 µL loaded, replicate #1 (12) SUMO-PylB, 2 min digest, 8 µL loaded, replicate #1 (13) PylB.3f2, 2 min digest, 8 µL loaded, replicate #1 (14) PylB.JM10.1, 2 min digest, 8 µL loaded, replicate #1


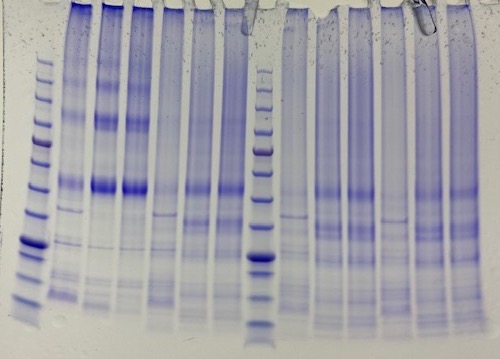


**Chymotrypsin proteolysis gel image #2**. Samples loaded in each lane (from left to right) are: (1) Bluestain 2 Protein Ladder, 4 µL loaded (2) SUMO-PylB, undigested, 8 µL loaded, replicate #1 (3) PylB.3f2, undigested, 8 µL loaded, replicate #1 (4) PylB.JM10.1, undigested, 8 µL loaded, replicate #1 (5) SUMO-PylB, 5 minute digest, 8 µL loaded, replicate #1 (6) PylB.3f2, 5 minute digest, 8 µL loaded, replicate #1 (7) PylB.JM10.1, 5 minute digest, 8 µL loaded, replicate #1 (8) Bluestain 2 Protein Ladder, 4 µL loaded (9) SUMO-PylB, 10 min digest, 8 µL loaded, replicate #1 (10) PylB.3f2, 10 min digest, 8 µL loaded, replicate #1 (11) PylB.JM10.1, 10 min digest, 8 µL loaded, replicate #1 (12) SUMO-PylB, 15 min digest, 8 µL loaded, replicate #1 (13) PylB.3f2, 15 min digest, 8 µL loaded, replicate #1 (14) PylB.JM10.1, 15 min digest, 8 µL loaded, replicate #1


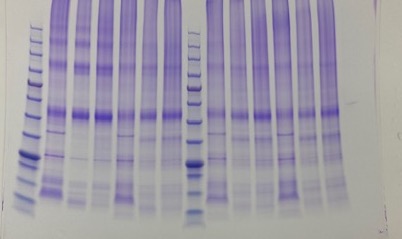


**Chymotrypsin proteolysis gel image #3**. Samples loaded in each lane (from left to right) are: (1) Bluestain 2 Protein Ladder, 4 µL loaded (2) SUMO-PylB, undigested, 8 µL loaded, replicate #2 (3) PylB.3f2, undigested, 4 µL loaded, replicate #2 (4) PylB.JM10.1, undigested, 5 µL loaded, replicate #2 (5) SUMO-PylB, 30 second digest, 8 µL loaded, replicate #2 (6) PylB.3f2, 30 second digest, 4 µL loaded, replicate #2 (7) PylB.JM10.1, 30 second digest, 5 µL loaded, replicate #2 (8) Bluestain 2 Protein Ladder, 4 µL loaded (9) SUMO-PylB, 1 min digest, 8 µL loaded, replicate #2 (10) PylB.3f2, 1 min digest, 4 µL loaded, replicate #2 (11) PylB.JM10.1, 1 min digest, 5 µL loaded, replicate #2 (12) SUMO-PylB, 2 min digest, 8 µL loaded, replicate #2 (13) PylB.3f2, 2 min digest, 4 µL loaded, replicate #2 (14) PylB.JM10.1, 2 min digest, 5 µL loaded, replicate #2


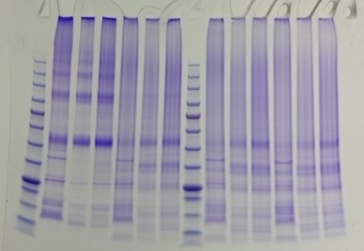


**Chymotrypsin proteolysis gel image #4**. Samples loaded in each lane (from left to right) are: (1) Bluestain 2 Protein Ladder, 4 µL loaded (2) SUMO-PylB, undigested, 8 µL loaded, replicate #2 (3) PylB.3f2, undigested, 4 µL loaded, replicate #2 (4) PylB.JM10.1, undigested, 5 µL loaded, replicate #2 (5) SUMO-PylB, 5 min digest, 8 µL loaded, replicate #2 (6) PylB.3f2, 5 min digest, 4 µL loaded, replicate #2 (7) PylB.JM10.1, 5 min digest, 5 µL loaded, replicate #2 (8) Bluestain 2 Protein Ladder, 4 µL loaded (9) SUMO-PylB, 10 min digest, 8 µL loaded, replicate #2 (10) PylB.3f2, 10 min digest, 4 µL loaded, replicate #2 (11) PylB.JM10.1, 10 min digest, 5 µL loaded, replicate #2 (12) SUMO-PylB, 15 min digest, 8 µL loaded, replicate #2 (13) PylB.3f2, 15 min digest, 4 µL loaded, replicate #2 (14) PylB.JM10.1, 15 min digest, 5 µL loaded, replicate #2


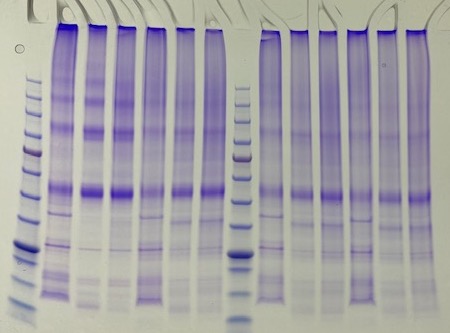


**Chymotrypsin proteolysis gel image #5**. Samples loaded in each lane (from left to right) are: (1) Bluestain 2 Protein Ladder, 4 µL loaded (2) SUMO-PylB, undigested, 8 µL loaded, replicate #3 (3) PylB.3f2, undigested, 4 µL loaded, replicate #3 (4) PylB.JM10.1, undigested, 5 µL loaded, replicate #3 (5) SUMO-PylB, 30 second digest, 8 µL loaded, replicate #3 (6) PylB.3f2, 30 second digest, 4 µL loaded, replicate #3 (7) PylB.JM10.1, 30 second digest, 5 µL loaded, replicate #3 (8) Bluestain 2 Protein Ladder, 4 µL loaded (9) SUMO-PylB, 1 min digest, 8 µL loaded, replicate #3 (10) PylB.3f2, 1 min digest, 4 µL loaded, replicate #3 (11) PylB.JM10.1, 1 min digest, 5 µL loaded, replicate #3 (12) SUMO-PylB, 2 min digest, 8 µL loaded, replicate #3 (13) PylB.3f2, 2 min digest, 4 µL loaded, replicate #3 (14) PylB.JM10.1, 2 min digest, 5 µL loaded, replicate #3


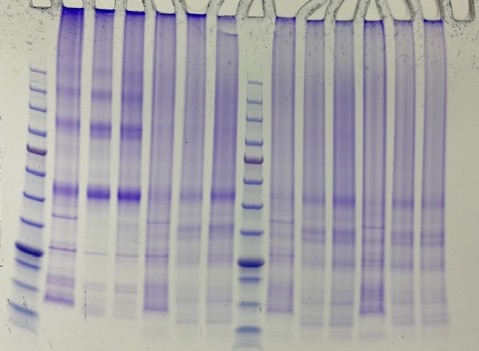


**Chymotrypsin proteolysis gel image #6**. Samples loaded in each lane (from left to right) are: (1) Bluestain 2 Protein Ladder, 4 µL loaded (2) SUMO-PylB, undigested, 8 µL loaded, replicate #3 (3) PylB.3f2, undigested, 4 µL loaded, replicate #3 (4) PylB.JM10.1, undigested, 5 µL loaded, replicate #3 (5) SUMO-PylB, 5 min digest, 8 µL loaded, replicate #3 (6) PylB.3f2, 5 min digest, 4 µL loaded, replicate #3 (7) PylB.JM10.1, 5 min digest, 5 µL loaded, replicate #3 (8) Bluestain 2 Protein Ladder, 4 µL loaded (9) SUMO-PylB, 10 min digest, 8 µL loaded, replicate #3 (10) PylB.3f2, 10 min digest, 4 µL loaded, replicate #3 (11) PylB.JM10.1, 10 min digest, 5 µL loaded, replicate #3 (12) SUMO-PylB, 15 min digest, 8 µL loaded, replicate #3 (13) PylB.3f2, 15 min digest, 4 µL loaded, replicate #3 (14) PylB.JM10.1, 15 min digest, 5 µL loaded, replicate #3
